# Emergent Nanostructure and Ion Transport in Polyzwitterion/Polyanion Blends

**DOI:** 10.1021/acs.macromol.5c00806

**Published:** 2025-08-13

**Authors:** Hongwei Li, Qinyu Zhu, Yuya Shinohara, Yangyang Wang, Panagiotis Christakopoulos, Autumn F. Kudlack, Zitan Huang, Peter V. Bonnesen, Changwoo Do, Md Anisur Rahman, Michelle L. Lehmann, Tomonori Saito, Ralph H. Colby, Rajeev Kumar, Jodie L. Lutkenhaus

**Affiliations:** † Artie McFerrin Department of Chemical Engineering, 14736 Texas A&M University, College Station, Texas 77843, United States; ‡ Center for Nanophase Materials Sciences, 6146 Oak Ridge National Laboratory, Oak Ridge, Tennessee 37831, United States; § Materials Science and Technology Division, Oak Ridge National Laboratory, Oak Ridge, Tennessee 37831, United States; ∥ Department of Materials Science and Engineering, The Pennsylvania State University, University Park, Pennsylvania 16802, United States; ⊥ Neutron Scattering Division, Oak Ridge National Laboratory, Oak Ridge, Tennessee 37831, United States; # Chemical Sciences Division, Oak Ridge National Laboratory, Oak Ridge, Tennessee 37830, United States; ∇ Department of Materials Science and Engineering, Texas A&M University, College Station, Texas 77840, United States

## Abstract

Solid polymer electrolytes (SPEs) hold great promise for the advancement of next-generation energy storage devices. However, the ion transport mechanism in SPEs remains poorly understood. In this study, we investigate blends of poly­(1-(3-sulfonatopropyl)-2-vinylpyridinium) (P2VPPS) and poly­(lithium (trifluoromethane)­sulfonimide methacrylate) (P­(MTFSI)­Li) of varying molar ratios to develop a mechanistic understanding of ionic conductivity in a miscible polyzwitterion/polyanion system. Polyanions can act as single-ion conductors, but conductivity is often prohibitively low due to the decreased segmental mobility and ion aggregation. Here, it is hypothesized that the introduction of a polyzwitterion would competitively interact with the polyanion charge groups to realize improvements in the conductivity. Attractive interactions between the polyanions and polyzwitterions are confirmed by the blend’s increased glass transition temperature using the Gordon–Taylor equation. Notably, an ordered local nanostructure (∼24 Å) emerged in the P2VPPS/P­(MTFSI)Li system at certain compositions, as characterized by small-angle X-ray and neutron scattering (SAXS/SANS). Concurrent with the emergence of this structure, broadband dielectric spectroscopy confirmed improvements in ionic conductivity. The highest conductivity is observed at a specific blend ratio P2VPPS:P­(MTFSI)­Li = 0.2:1 in the glassy state and 0.3:1 in the rubbery state, corresponding to the lowest effective activation energy (E*). Coarse-grained molecular dynamics simulations further emphasize the role of complexation between polyzwitterion and polyanion chains, correlating with the emergence of a new peak in SAXS and SANS for the blends. This work provides a fresh perspective on the role of local structural design in developing SPEs and offers insights into the morphological effects on ionic conductivity.

## Graphical abstract

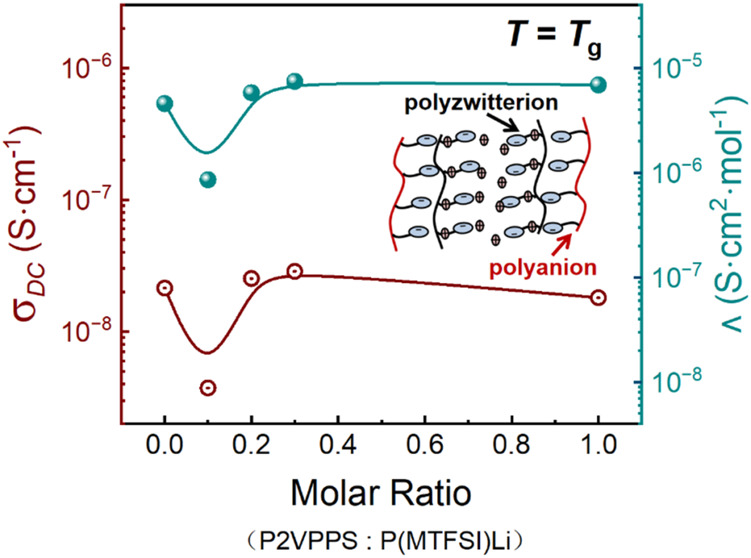

## Introduction

Solid polymer electrolytes (SPEs) have been under extensive scrutiny due to technological and academic interests in developing the next generation of batteries and structure–property relations.^1–9^ Compared to liquid electrolytes and inorganic solid electrolytes, SPEs are nonvolatile, are flame resistant, and exhibit good processability.^7–9^ Different types of SPEs have been developed and studied, including single-ion conducting polymers such as polymerized ionic liquids, block copolymer electrolytes, and composites containing nanoparticles and polymers.^1–6^ Currently, the relatively low conductivity and limited understanding of ion conduction hinder the commercial applications of SPEs.^8,10^ There is a worldwide effort focused on enhancing the ionic conductivity of SPEs, especially near room temperature, by manipulation of various characteristics discussed below.

In recent years, polymer electrolyte blends have been studied with the aim of improving ionic conductivity.^3,11–14^ These studies are motivated by three main physical principles: (1) lowering the glass transition of the blends should lead to faster dynamics near room temperature; (2) an increase in static dielectric constant of the polymer matrix should reduce the energy barrier for ion transport, which should, in turn, lead to enhanced charge transport; and (3) enhancing decoupling of charge transport from segmental dynamics of polymers can lead to enhanced conductivity. For example, poly­(ethylene oxide) and polycarbonates have been blended with either lithium salt or polymerized ionic liquids.^3,11,15^ These studies have shown plasticization effects of the additives; i.e., a reduction of the glass transition temperature led to an enhancement of isothermal ionic conductivity. In addition, either the use of polar additives or the introduction of polar groups on monomers for the purpose of increasing the static dielectric constant of blended polymers has exhibited competing effects between ionic aggregation and segmental mobility.^16–18^ In particular, the DC conductivity has been shown to first increase and then decrease with an increase in the static dielectric constant. The nonmonotonic behavior of the conductivity on increasing polarity of monomers has been shown to result from the breakup of ionic clusters, leading to an increase in the conductivity, followed by the slowing down of matrix dynamics, which causes the conductivity to decrease.^18^ Despite the counteracting effects resulting from the simultaneous increase in the static dielectric constant and the glass transition temperature for the system in ref 18, it has been demonstrated that the ionic conductivity in blends of polyzwitterions and lithium salts can increase relative to the polyzwitterion alone.^6,19^

Furthermore, several studies suggest that incorporating polar or π-conjugated groups into polymer electrolytes could enhance ion transport by facilitating the formation of favorable local structures.^20–22^ Polar groups that can participate in hydrogen bonding can weaken the electrostatic interactions between Li^+^ and anions while influencing the segmental arrangement of the conducting polymer, ultimately improving conductivity.^20^ Specifically, a polymer class featuring hydrogen-bonded moieties exhibited promising ionic conductivities with low activation energy upon lithium salt addition, as these moieties directed and facilitated the formation of a long-range-ordered lamellar nanostructure.^23^ Similarly, in poly­(ethylene oxide) (PEO)-based systems, local structure was formed through interactions between PEO and an α-cyclodextrin additive.^24^ This structure facilitated Li^+^ ion transport while preventing anion access via size exclusion. Moreover, high ionic conductivity and the formation of local aggregates have been observed in cationic conjugated polyelectrolytes with imidazolium groups, attributed to the tunable ionic interaction strength and functionalized cationic side chain that facilitate π^+^–π^+^ stacking.^22^ This further highlights the impact of long-range polymer ordering as well as polymer–ion and ion–ion interactions on ionic conductivity.

We are aware of two studies reporting enhancement of ionic conductivities due to the addition of polyzwitterions.^6,19^ In both reports, a strong decoupling of ion transport from the matrix dynamics is present. However, the origin of the decoupling is not clear. In this work, we have explored the use of polyzwitterions in enhancing the ionic conductivity of single-ion conducting polymer electrolytes. Our main aim is to develop a mechanistic understanding of the ionic conductivity in these blends while studying the effects of the changes in the glass transition temperature and local structure for different mixing ratios. We observe nonmonotonic behavior of the ionic conductivity with an increase in the polyzwitterion content in the blends. The conductivity first decreases and then increases, before decreasing again, with an increase in the polyzwitterion content in the blends. These changes in DC conductivity are tracked with the changes in the local structure of the blends and probed using small-angle X-ray and neutron scattering (SAXS and SANS). Coarse-grained molecular dynamics simulations are used to interpret changes in the local structure of the blends, which highlights the role of complexation among polyzwitterion and polyanion chains, leading to the emergence of a new peak in the SAXS and SANS. These changes in local structure are conjectured to be responsible for the nonmonotonic changes in the ionic conductivity of the blends.

## Materials and Methods

### Materials

The polyzwitterion poly­(1-(3-sulfonatopropyl)-2-vinylpyridinium) (P2VPPS) with number-average molecular weight, (*M*_*n*_)=14,500 g/mol, and dispersity, *M*_*w*_ */M*_*n*_ = *Đ* = 1.89 (Figure 1a), was synthesized using free radical polymerization techniques and is described elsewhere.^25^ Briefly, in a typical run, 10 g of 1-(3-sulphonatopropyl)-2-vinylpyridinium was dissolved in H_2_O along with the initiator (0.055 g of K_2_S_2_O_8_) and purged with Argon before heating in an oil bath at 343 K for 24 h to obtain the final polymer.

**Figure 1:**
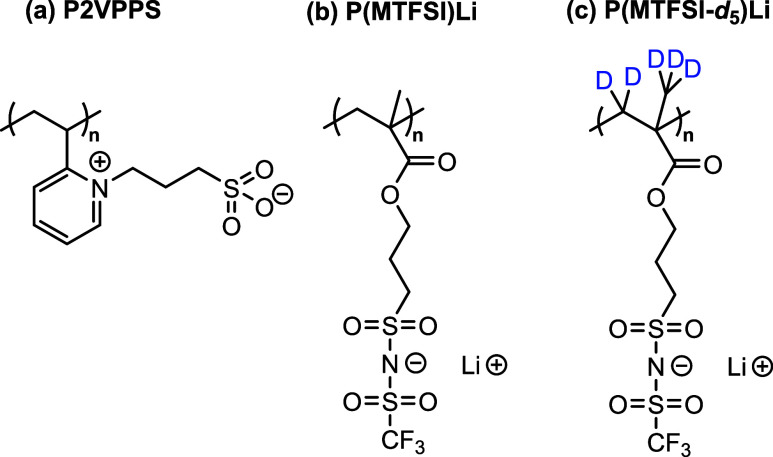
Chemical structures of (a) poly­[1-(3-sulphonatopropyl)-2-vinylpyridinium] (P2VPPS), (b) poly­(methacrylate trifluoromethane sulfonimide lithium) [P­(MTFSI)­Li], and (c) poly­(methacrylate trifluoromethane sulfonimide lithium*-d*_5_) [P­(MTFSI-*d*_5_)­Li].

The number-average molecular weights (*M*_*n*_) and dispersity *Đ* (*M*_w_/*M_n_*) were measured using SEC for P2VPPS. Samples were carried out using an Agilent 1260 Infinity LC stack (pump, vacuum degasser, and autosampler) pumping system with H_2_O containing 0.1 M NaNO_3_ as the eluent at 1 mL min^–1^, a Phenomenex ThermaSphere HPLC column heater/chiller at 313 K, and dual Wyatt detectors (miniDawn TREOS light-scattering detector and an Optilab rEX refractive index detector at 313 K). The columns used for the separation were 2 Agilent PLgel MIXED-C (7.5 mm ID × 300 mm, 5 μm). Absolute molar masses were calculated using bovine serum albumin (BSA) at 67 kDa as the narrow standard and β-lactoglobulin at 35 kDa as the broad standard.

The polyanion, poly­(lithium (trifluoromethane)­sulfonimide methacrylate) (P­(MTFSI)­Li), with *M*_*n*_ = 25,000 g/mol and *Đ* = 1.80, shown in Figure 1b, was prepared following a previously reported procedure.^26^ 1.0 g (2.898 mmol) of (trifluoromethane)­sulfonimide lithium methacrylate (LiMTFSI) (from Specific Polymers, used as received) and 4.75 mg (0.0289 mmol) of azobis­(isobutyronitrile) (AIBN) were dissolved in 2 mL of anhydrous *N,N*-Dimethylformamide (DMF) with stirring under an inert atmosphere (glovebox) at room temperature in a 10 mL Schlenk flask. The clear solution was then polymerized under an inert atmosphere at 348 K overnight. The resulting viscous polymer solution was diluted with Milli-Q water (18 MΩ, 3–5 mL), then dialyzed against water for 3 days (water was changed at least 3 times during dialysis). The dialyzed solution was rotavapped to remove all of the solvents to collect pure P­(MTFSI)Li and further dried under a high vacuum at 393 K for 1 day.

Lithium ((3-((2-(methyl-*d*_3_)­acryloyl-*d*_2_)­oxy)­propyl)­sulfonyl)­((trifluoromethyl)-sulfonyl)­amide (MTFSI Li-*d*_5_) was synthesized in a stepwise procedure starting with commercially obtained methyl-*d*_3_ methacrylate-*d*_5_, as described in the Supporting Information section. Polymerization of P­(MTFSI-*d*_5_)Li (*M*_*n*_ = 26 100 g/mol, *Đ* = 1.90) followed a similar procedure as P­(MTFSI)­Li, except that methanol was utilized as the dialyzing solution instead of Milli-Q water (see Figure 1c for the chemical structure).,

The molecular weight and polydispersity of P­(MTFSI)Li and P­(MTFSI-*d*_5_)Li were measured with a Malvern OMNISEC GPC system (Malvern Panalytical Ltd.) equipped with OMNISEC RESOLVE and OMNISEC REVEAL (Malvern Panalytical Ltd.). The sample concentrations were 8 mg mL^–1^, and each was filtered through 30 mm 0.45 μm polytetrafluoroethylene (PTFE) filters prior to analysis. The analysis was carried out using two PLgel 5 μm mixed-C columns (7.5 mm ID × 300 mm) in series. Dimethylformamide (DMF) with 0.05 M lithium bromide (LiBr) was utilized as the eluent (0.4 mL min^–1^, with the entire system at 333 K). Absolute molar masses were calculated relative to poly­(methyl methacrylate) (PMMA) standards in the OMNISEC software v5.10.

### Preparation of Blends

Briefly, the synthesized P2VPPS and P­(MTFSI)Li were first dissolved in Milli-Q water at a concentration of 100 mM (by repeat unit) with a resistivity of 18.2 MΩ·cm. Then the polyzwitterion/polyanion blends were prepared by mixing P2VPPS and P­(MTFSI)Li aqueous solutions at various molar ratios (P2VPPS:P­(MTFSI)­Li = 0.1:1, 0.2:1, 0.3:1 and 1:1). To remove the solvent (water) and obtain the solid P2VPPS/P­(MTFSI)Li blends, we evaporated the free water from the mixture solutions at 343 K for 1 day. Subsequently, the predried solid blends were moved into a vacuum oven at 393 K overnight for further drying. Finally, these completely dried blends were used for the following experimental measurements.

### Differential Scanning Calorimetry (DSC)

The glass transition temperatures were characterized for neat P­(MTFSI)­Li, neat P2VPPS, and their blends with various mixing ratios (P2VPPS:P­(MTFSI)Li = 0.1:1, 0.2:1, 0.3:1, and 1:1) using DSC (Q200, TA Instruments). All neat polymers or blends were dried at room temperature for 1–2 days to remove free water and further dried at 393 K overnight in a vacuum oven. Immediately after, samples of 4–10 mg dry powder were sealed into Tzero pans with lids and went through at least two cooling and heating cycles at 10 K/min, ramped from 313 to 503 K. The second heating cycle was used to determine the *T*_g_ values.

### Thermogravimetric Analysis (TGA)

The thermal decomposition was characterized using TGA (TA Instruments, TGA 5500) at a heating rate of 10 K/min from 298 to 873 K under a flowing nitrogen atmosphere. 3.0–5.0 mg samples were added to platinum HT pans. The decomposition temperature (*T*_d_) was determined from the point on the curve (in Figure S1) where 95 wt % of mass loss (based on the onset of plateau) had occurred after solvent (water) evaporation. The *T*_d_’s were 537 and 562 K for P2VPPS and P­(MTFSI)­Li, respectively.

### Broadband Dielectric Spectroscopy (BDS)

Ionic conductivity and dielectric relaxations were determined by BDS using an α–A analyzer (Novocontrol) with 0.1 V excitation amplitude and ZGS test interface in the frequency range of 10^–1^ to 10^7^ Hz. The sandwiched polymer–electrode samples were prepared in a sample-cell assembly with a ceramic holder, where the dry P2VPPS/P­(MTFSI)Li blends were hot pressed into a film in a convection oven of a Discovery HR-2 rheometer (TA Instruments) at approximately 20 K above *T*_g_ under nitrogen gas protection. The thickness, area, and mass of blend films were used to convert the measured complex impedance to material-specific properties such as complex permittivity and molar conductivity. The experiments were performed every 10 K (and every 5 K near the glass transition) in a nitrogen atmosphere from low to high temperatures, with 20 min equilibration time prior to each measurement. We were unable to do measurements upon cooling due to testing the sample close to the polymers’ *T*_d_’s and the samples’ slight yellowing.

### X-Ray Scattering

Small-angle X-ray scattering (SAXS) and wide-angle X-ray scattering (WAXS) were measured using a Xenocs Xeuss 3.0 instrument equipped with a D2+ MetalJet X-ray source (Ga Kα). Polyimide films were used for the windows of each sample cell, and the nominal thickness of each sample was 1.0 mm. The effective thickness, taking into account the packing ratio of the sample powder, was estimated by using the X-ray transmission and the density of each sample. The sample temperature was controlled by a temperature-control stage (DSC600, LINKAM). The scattered beam was recorded using a Dectris Eiger 2R 4 M Hybrid photon counting detector. The transmission of X-rays was also observed by using the same detector without a beam stop, making it possible to convert the measured intensity into the absolute-scattering intensity. The exposure time was 600 and 300 s for SAXS and WAXS, respectively. The collected SAXS and WAXS images were circularly averaged and converted to one-dimensional scattering intensity profiles, *I*(*q*), where *q* is the magnitude of the scattering vector. The sample-to-detector distance was 900 and 88.8 mm for SAXS and WAXS, respectively. After converting to the absolute-scattering intensity, SAXS and WAXS were merged as a single-scattering profile.

### Neutron Scattering

Small-angle neutron scattering (SANS) was performed at the EQ-SANS (SNS, BL-6) instrument at the Spallation Neutron Source (SNS), Oak Ridge National Laboratory.^27,28^ All samples were loaded into a 0.5 mm thick demountable Ti cell, where they were sandwiched between two quartz windows. Measurements were conducted at a sample-to-detector distance of 1.3 m, using a wavelength band with a minimum wavelength of 1 Å, providing a *q*-range of 0.01 to 2.5 Å^–1^. Data reduction followed the standard procedure outlined in the reference, incorporating corrections for detector sensitivity and empty-cell background subtraction.^29,30^ Sample temperature was varied and maintained using a Peltier block sample holder with a minimum equilibration time of 20 min to ensure thermal stability. The method of sample preparation was similar to that for DSC. The ratios were kept the same for reproducibility of the results. P2VPPS and P­(MTFSI)Li aqueous solutions at various molar ratios (0.1:1, 0.2:1, 0.3:1, and 1:1) were prepared by mixing aqueous solutions of the respective polymers. Subsequently, the solutions were freeze-dried, obtaining solid samples, which were then put in the SANS cells and allowed to dry overnight in a vacuum oven at 393 K to remove all residual solvent.

### Coarse-Grained Molecular Dynamics Simulations

A series of coarse-grained molecular dynamics simulations (CGMD) were performed using the Large Scale Atomic/Molecular Massively Parallel Simulator (LAMMPS) package.^31^ The simulation systems consisted of blends of polyanions (PA) and polyzwitterions (PZ) at different mixing molar ratios (PZ:PA = 0.1, 0.2, and 0.3, respectively). The coarse-grained models of PA and PZ chains are shown in Figure S2. Each chain contained 35 beads in the backbone, with all beads having a uniform size of 1 σ, except for the counterion beads, which have a size of 0.22 σ and represent a lithium cation of radius 0.073 nm for σ = 0.33 nm, approximately twice the carbon–carbon bond length.^32^ All short-range pairwise interactions were described by the truncated Lennard-Jones (LJ) potential: $U_{\mathrm{LJ}}\left(r_{\mathrm{ij}}\right)=4\epsilon \left[{\left(\frac{\sigma }{r_{\mathrm{ij}}}\right)}^{12}-{\left(\frac{\sigma }{r_{\mathrm{ij}}}\right)}^{6}\right],\;r_{ij}<r_{\mathrm{cut}}$ , where ϵ = 1 *k*_B_ *T* and *r*_cut_ = 2.5 σ for all pairwise interactions, and we use r_ij_ to represent the norm of **r**_ij_. The bond connectivity in the polymer chains was characterized using finite extensible nonlinear elastic (FENE) potential: $U_{\mathrm{FENE}}\left(r_{\mathrm{ij}}\right)=-\frac{1}{2}k_{b}R_{0}^{2}\;\ln\left[1-{\left(\frac{r_{\mathrm{ij}}}{R_{0}}\right)}^{2}\right]$ , with *k*_b_ = 30 ϵ/σ^2^ and *R*_0_ = 1.5σ.^33^ The electrostatic interactions between charged species with charges q_*i*_ and q_*j*_ were modeled using the Coulomb potential: $U_{Coulomb}(r_{ij})=\frac{q_{i}q_{j}}{4\pi \epsilon_{0}\epsilon_{r}r_{\mathrm{ij}}},\;r_{\mathrm{ij}}<r_{cut,\mathrm{elec}}$ , where ϵ_0_ is the permittivity of vacuum and ϵ_*r*_ is the relative permittivity of the polymer matrix, which we set to 2 to mimic the properties of the hydrocarbon backbone environment, and the dimensionless charge |q_i_|=|q_j_|=q^*^=q/√4πε_0_ϵσ. In order to simulate monovalent charges on the chains and monovalent counterions, was taken to be 7.48 under the assumption that q = 1.6 × 10^–19^ C, σ = 1 nm and ϵ/k_B_ = 298 K. An electrostatic cutoff distance *r*_elec,cut_ of 8 σ was used for balanced accuracy and efficiency of the CGMD simulations.

The initial conditions were generated by purposefully placing monomer beads using self-avoiding random walks. The counterions of the polyanions were then distributed randomly in the simulation box. To eliminate overlaps or unfavorable initial configurations in the polymer chains, the systems were first relaxed in the microcanonical (NVE) ensemble with constraints on the maximum displacement per time step. Following the initial energy minimization, the simulations were then performed in isothermal–isobaric (NPT) conditions at dimensionless T* = *k*_B_ *T*/ϵ = 1.0 and $P{\;}^{*}=P\frac{\sigma^{3}}{\epsilon }=0$ . All simulations were performed in periodic boundary conditions in all dimensions with a constant time step Δ*t* = 0.005 τ_LJ_ (τ_LJ_: LJ time). The systems were first equilibrated for 5000 τ_LJ_ to ensure that the time evolutions of thermodynamic quantities (e.g., temperature, pressure, total energy, etc.) reached a steady state. The structure factor results were calculated as the time-average based on the trajectory files from *t* = 5000 τ_LJ_ to *t* = 25,000 τ_LJ_. Partial structure factors calculated from the backbone beads of the polyanions were used to interpret the SANS results (see the definitions of variables in the Abbreviations section.)

## Results and Discussion

### Glass Transitions of Dry P2VPPS/P­(MTFSI)Li Blends

DSC was used to examine the thermal behavior of neat P2VPPS, neat P­(MTFSI)­Li, and their blends, as shown in Figure S3. The *T*_g_ values were determined from the inflection point in the sigmoidal region of the thermogram. Specifically, the *T*_g_ values ranged from 459 to 479 K as marked by the short vertical lines in Figure S3. Neat P­(MTFSI)Li exhibited a *T*_g_ of 457 K, which was lower than that of neat polyzwitterion, P2VPPS (*T*_g_ = 499 K). As for the blends, each one displayed a single glass transition and no other thermal features, indicating that the two polymers were miscible and that no crystallinity was present.^34,35^ As the P2VPPS content increased in the blend, *T*_g_ increased but remained between that of the two homopolymers as the mixing ratio of P2VPPS/P­(MTFSI)­Li increased from 0.1:1 to 1:1. This result suggests that the motion of P­(MTFSI)Li chains is more restricted in blends as compared to the neat polyanion. Given the relatively high *T*_g_’s of the polymers and the blends, as well as the *T*_d_’s of the neat polymers (537 K for neat P2VPPS and 562 K for neat P­(MTFSI)­Li, respectively, Figure S1), thermal analysis of blends with higher P2VPPS content was restricted to 503 K maximum.

For the P2VPPS/P­(MTFSI)­Li blends, the change in *T*_g_ values can be described by simple mixing rules. One rule employed to describe the blends’ *T*_g_’s is calculated by the Fox equation^36^ (orange dashed line in Figure 2):

1 $$\frac{1}{T_{g}}=\frac{w_{1}}{T_{g,1}}+\frac{w_{2}}{T_{g,2}}$$

where *T*_g_ is the glass transition temperature of the blend; *w*_1_ and *w*_2_ are the weight fractions of each component, P­(MTFSI)Li and P2VPPS, respectively, in a blend. *T*_g,1_ and *T*_g,2_ are the *T*_g_ values of neat P­(MTFSI)Li and neat P2VPPS, respectively. Elsewhere, the Fox equation has been applied to interpret the observed *T*_g_ of dry polyelectrolyte complexes.^37^ Based on our calculations, the results demonstrate that the blends’ *T*_g_’s should increase as the P2VPPS content increases. The Fox equation is generally in agreement with experimental data from DSC (within a deviation of less than 10 K), but the Fox equation systematically underpredicted the blends’ experimental *T*_g_’s. Instead, the *T*_g_ of the miscible polymer blends was also examined by applying the Gordon–Taylor equation:^38,39^

2 $$T_{g}=\frac{w_{1}T_{g,1}+kw_{2}T_{g,2}}{w_{1}+kw_{2}}$$

where the fitting parameter *k* represents a constant related to the miscibility and intercomponent interactions in the blend, capturing deviations from an ideal mixture and the unequal contribution of each component to the overall mobility of the blend.^11,38,40–44^ In the P2VPPS/P­(MTFSI)Li blend, the best Gordon–Taylor fitting was obtained when taking a positive *k* value of 1.83, which manifests as a concave down curve and indicates attractive interactions between P2VPPS and P­(MTFSI)­Li, likely arising from binding between the polyzwitterion’s pyridinium group and the polyanion’s TFSI group. This binding is desirable because we expect that it will weaken the interaction between the lithium cation and the polyanion’s TFSI group. In contrast, smaller values of *k* (<1), resulting in concave up *T*_g_-composition curves, have been reported for PEO-based and polyethylene glycol (PEG)-based single-ion conductors.^11,45,46^

**Figure 2:**
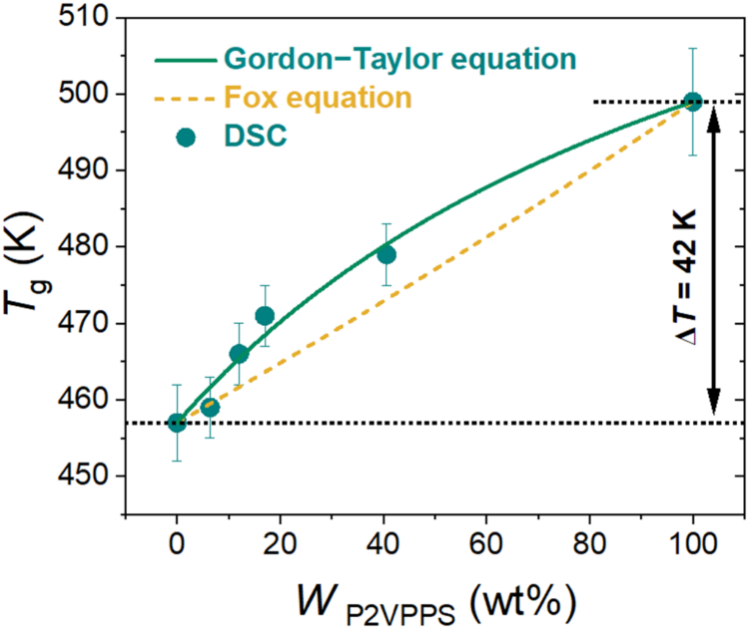
Glass transition temperature (*T*_g_) of neat P2VPPS, neat P­(MTFSI)­Li, and their blends was determined from DSC. The dashed line corresponds to the Fox equation, and the solid line corresponds to the Gordon–Taylor equation fit using eqs 1 and 2, respectively.

### Local Structures of Blends

Figure 3 shows the absolute X-ray scattering and small-angle neutron scattering (SANS) intensity profiles, *I*(*q*), of neat P2VPPS, neat P­(MTFSI)­Li, and blends of P2VPPS/P­(MTFSI)Li at room temperature. The profiles are offset vertically by 0.05 cm^–1^ (Figure 3a) and 0.1 cm^–1^ (Figure 3b), respectively, for the sake of clarity. In Figure 3a, neat P­(MTFSI)Li and neat P2VPPS show a major positive correlation peak at *q* = 1.2 and 1.35 Å^–1^, respectively, corresponding to the amorphous halo of the neat polymer chains.^47,48^ The peak height of P­(MTFSI)Li is higher than that of P2VPPS, likely because the side chain of P­(MTFSI)Li contains more heavy ions (sulfur) than does P2VPPS, leading to a larger scattering contrast. At a similar *q*, the blends also show a positive correlation peak (*q* = 1.19 Å^–1^), which is approximated by the weighted sum of *I*(*q*) of those pure samples (Figure S4) using their molar ratio. Meanwhile, a new positive correlation peak associated with pairs of P2VPPS/P­(MTFSI)Li at 0.26 Å^–1^ appears when the blend ratio of P2VPPS was larger than 0.3:1. X-ray scattering was also conducted at 423 K, which is still at conditions below the blends’ *T*_g_’s, Figure S5a. The new peak shifted to a smaller *q* value of 0.25 Å^–1^ and became slightly more pronounced, which can be attributed to thermal expansion.^49^ The emergence of this new low *q* peak cannot be explained by the combination of the scattering intensity profiles of neat P2VPPS and P­(MTFSI)­Li, which indicates the formation of a nanostructure in the blend. This nanostructure has a periodicity of 24–25 Å defined by 2π/*q*, which may be associated with the ordering of the polymers, as discussed below.

**Figure 3:**
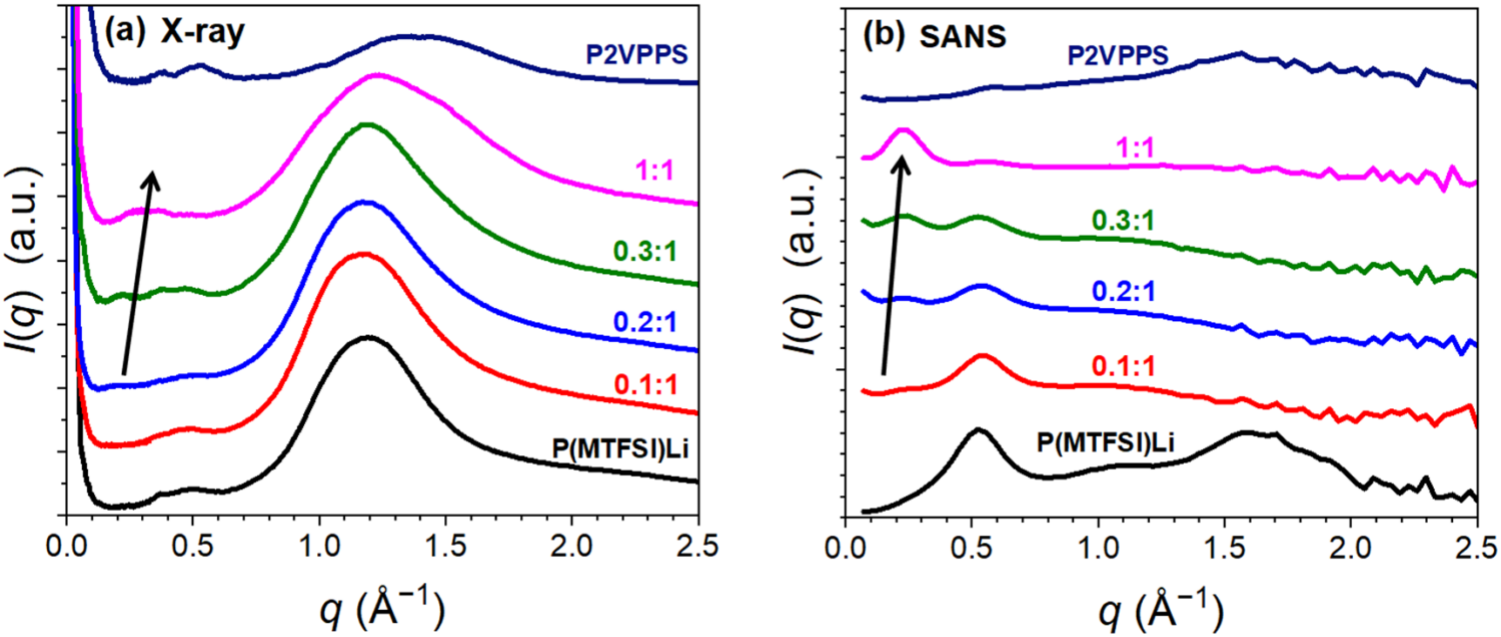
Scattering profiles of homopolymers and blends of different composition ratios. (a) Absolute X-ray scattering and (b) small-angle neutron scattering (SANS) intensity profiles, *I*(*q*), of neat P2VPPS, neat P­(MTFSI)­Li, and their blends (P2VPPS:P­(MTFSI)­Li = 0.1:1, 0.2:1, 0.3:1, and 1:1) at room temperature. The intensity profiles were offset vertically by 0.05 and 0.1 cm^–1^, respectively. A positive correlation peak emerges at around 0.26 Å^–1^ as the ratio of PZ in the blend is increased, as shown by the arrows.

To further confirm the nanostructure at low *q* (0.2 to 0.3 Å^–1^), SANS was performed at room temperature for neat P2VPPS, neat P­(MTFSI-*d*_5_)­Li, and their blends. It should be noted that the P­(MTFSI-*d*_5_)Li used for SANS was deuterated at the backbone to highlight backbone–backbone correlations, Figure S6b,c. The deuteration process is described in the Supporting Information. The SANS signatures in Figure 3b show that, in addition to the amorphous halo, neat P­(MTFSI)Li exhibits correlations between polymer chains, at *q* = 0.5 Å^–1^, which weaken with the increasing P2VPPS content. At *q* = 0.2–0.3 Å^–1^, the peaks of the blends become more distinct and increase in intensity with rising P2VPPS content, suggesting the formation of a more ordered local structure. This is consistent with the low *q* values observed in X-ray scattering. These characteristic peaks appear only in the blends and are absent in the neat polymers, further indicating that the ordered local structure arises from the interaction between P2VPPS and P­(MTFSI)­Li.

The temperature-dependent effect (298, 333, and 393 K) observed with SANS is shown in Figure S5b, with no significant changes in the blends, which remained in a glassy state due to their high *T*_g_ values. The new scattering peak (*q* ≈ 0.26 Å^–1^) associated with the P2VPPS/P­(MTFSI)Li correlation persists at all temperatures (Figure S5), indicating that the polyzwitterion/polyelectrolyte correlation remains thermally stable up to 393 K (SANS) and 423 K (X-ray scattering). This thermal stability contributes to enhanced peak intensity. In literature reports of ionomer systems, the morphology observed at low *q* value in the X-ray scattering pattern is typically associated with ionic aggregation (20–60 Å), which disappears upon the addition of PEO.^11,50,51^ The suppression of ion aggregates is attributed to the plasticizing effect of PEO, which reduces the electron density and diminishes the scattering contrast between the polar and nonpolar phases. The intensified ionic aggregation peak upon heating can be explained by the randomization of dipole orientation and the decrease in dielectric constant with temperature, leading to enhanced ionic interactions between the ionic groups and the surrounding medium.^49,51^

For understanding the origin of the new scattering peak in the blends observed in Figure 3b, we performed CGMD simulations containing polyzwitterion and polyanion chains, each of the same length. For comparisons with the SANS experiments where P­(MTFSI-*d*_5_)­Li provided enhanced contrast from the backbones of the polyanions, we present the partial structure of the backbone beads belonging to polyanions in Figure 4a. Furthermore, structure factors of pure polyanions and polyzwitterions are also presented for comparison purposes. For the pure polyanions and polyzwitterions, two peaks were obtained in the partial structure factor for backbone–backbone correlations. In contrast, we found three distinct peaks in the structure factor for the blends, which is in qualitative agreement with the SANS experiments presented above. The high wavevector peak around |*q*| ∼7 σ^–1^ (labeled as peaks “1” in Figure 4a) arises from the distance between neighboring backbone beads within the same chain, which is commonly observed in CGMD simulations of polymer melts.^52^ The intermediate peak (labeled as “2”) around |*q*| ∼1.5 σ^–1^ corresponds to the distance between backbone beads on two chains driven by the attractive short-range LJ potentials and the electrostatic attraction between oppositely charged species in the side groups. Notably, these two peaks are not unique to the blends but are also present in the structure factors of the backbones in pure polyzwitterion and pure polyanion melts, which corresponds to the SAXS peak at around 0.5 Å^–1^ in Figure 3a. The peak is even more pronounced in the SANS data (see Figure 3b) at the same wavevector for the P­(MTFSI-*d*_5_)Li backbone. This suggests that ordered alignments and packing at similar length scales exist in both the pure melts and the blends.

**Figure 4:**
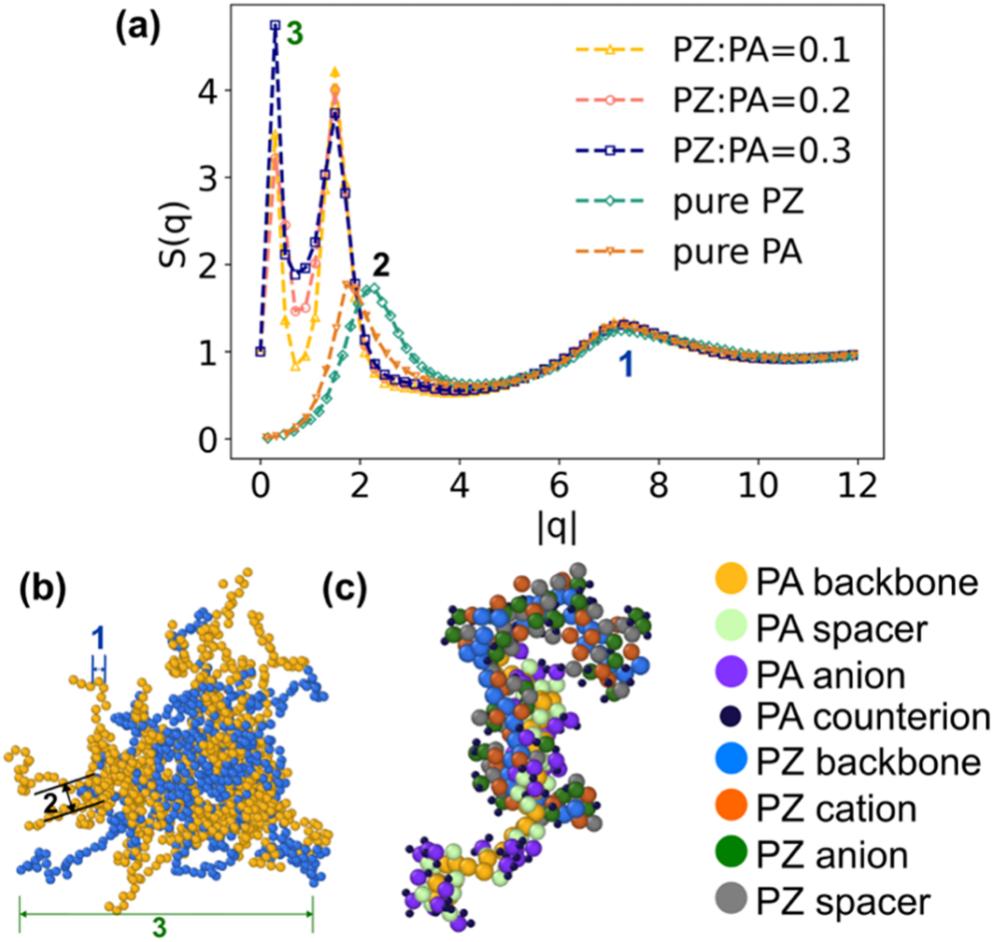
Summary of CGMD results. (a) Partial structure factor of backbone–backbone correlations for the polyanions in the blends at different mixing ratios, pure polyanions (PA), and pure polyzwitterions (PZ). Snapshots of a local cluster in a blend at a mixing ratio of PZ:PA = 0.3 that shows (b) only the backbone beads and (c) only the charged beads, which highlight the role of complexation in affecting the local structure in polyzwitterion-polyanion blends. Peaks observed in (a) correspond to the length scales represented in panel (b).

The new and the low-q peaks (labeled as peaks “3” in Figure 4a) are attributed to the size of the local clusters formed by the polyzwitterions and polyanions, as the dipoles on the polyzwitterion side chains and the pendant charges in the polyanions tend to form ionic complexes due to the strong electrostatic interactions. The intensity of this peak increases slightly with the increase in the PZ:PA ratio, as more of these local aggregates can form with the addition of a polyzwitterion in the system. The relative intensities of the first two peaks also align qualitatively with the SANS measurement in Figure 3b. Specifically, at PZ:PA ratios below 0.2, the intensity of the first peak is lower than that of the second, and at PZ:PA = 0.3, the intensities become comparable. Furthermore, we provide the simulation snapshots that visualize the local structures in polyzwitterion-polyanion blends shown in panels (b) and (c). These snapshots show that polyzwitterion chains tend to be miscible with polyanions due to complexation between them (see Figure 4b where only the backbones of PA and PZ are shown for clearer visualization), which is in qualitative agreement with experimental results presented here and elsewhere.^53^ We further isolated a single complexation pair of PA/PZ molecules along with the neighboring counterions (Figure 4c). The image clearly demonstrates how the chain segments are aligned due to the charge-dipole interactions in the pendant groups. In addition, the PZ molecules disrupt the interactions between the cations and the anions in the pure PA systems, as indicated by the small counterions that reside adjacent to the anions in the PZ molecules. To provide a more quantitative representation, we also present the radial distribution function of the lithium cation–PZ anion and the lithium cation–PA anion pairs in the Supporting Information (Figure S7), where we observe that real space correlations between the lithium cation and the anions of the PZ are weaker than those between the lithium cation and the anions of the PAs. The results suggest that the complexation and weaker interactions of the lithium cations with the anions of the PZ may facilitate faster cation transport in the PZ–PA blends.

It is important to point out that the *q* values at the peak locations do not exactly match the experimental results obtained from SANS when we rescale the structure factors to match the high *q* peak. Figure S8 shows a comparison of SANS and CGMD S­(q). This discrepancy is likely due to the differences between the coarse-grained molecular models used in the simulations and the real molecules studied in experiments. These differences, inherent in any CGMD simulation, arise from the simplified representation of molecular details and interactions. Nevertheless, the simulations still provide valuable insights into the origin of the new peak observed in the SANS data due to the complexation among polyzwitterion and polyanion chains, which is expected on the basis of charge-dipole interactions.

### Ionic Conductivity

BDS was employed next to understand the dielectric response and ionic conductivity of the blends. However, creating uniform films for BDS was challenging because the blends exhibited high *T*_g_ values, and thermal annealing was not easy because of the proximity of the decomposition temperature to the *T*_g_. Spin-coating and drop-casting methods were attempted, but the blends were brittle and prone to cracking and curling. Eventually, homogeneous films were obtained by hot-pressing P2VPPS/P­(MTFSI)Li blends of various molar ratios (0.1:1, 0.2:1, 0.3:1, and 1:1) in a ceramic holder. The BDS response for the neat polyzwitterion P2VPPS could not be obtained because of the film’s high *T*_g_ and its tendency to crack. See Figure S9 for the real part of the conductivity spectra obtained from the blends.

BDS was conducted from 353 to approximately 20 K above *T*_g_. At higher temperatures, we observed sample decomposition (film darkening), preventing us from using higher characterization temperatures. The DC conductivity (σ_DC_), presented in Figure 5a and normalized against *T*_g_ in Figure 5b, was extracted from the dielectric response (Figure S9) using the random barrier model (RBM).^26,54^ The molar conductivity was determined from σ_DC_ based on the lithium ion (Li^+^) concentration in the blend film, with the film’s volume calculated as detailed in the Supporting Information (Figure 5c,d). If the conductivity mechanism is similar for each of the blends, then the data should overlay each other into a single curve when normalized against *T*_g_. Figure 5 shows that the general shape of the conductivity-*T* responses is similar for all of the blends, but they do not overlay very well. Specifically, the overlay was best when *T*_g_/*T* was less than one or when the blend was rubbery, but there was a spread in the conductivity response when *T*_g_/*T* was greater than one or when the blend was glassy. This result indicates that the conductivity mechanism proceeds differently for the blends, especially in the blends’ glassy state.

**Figure 5:**
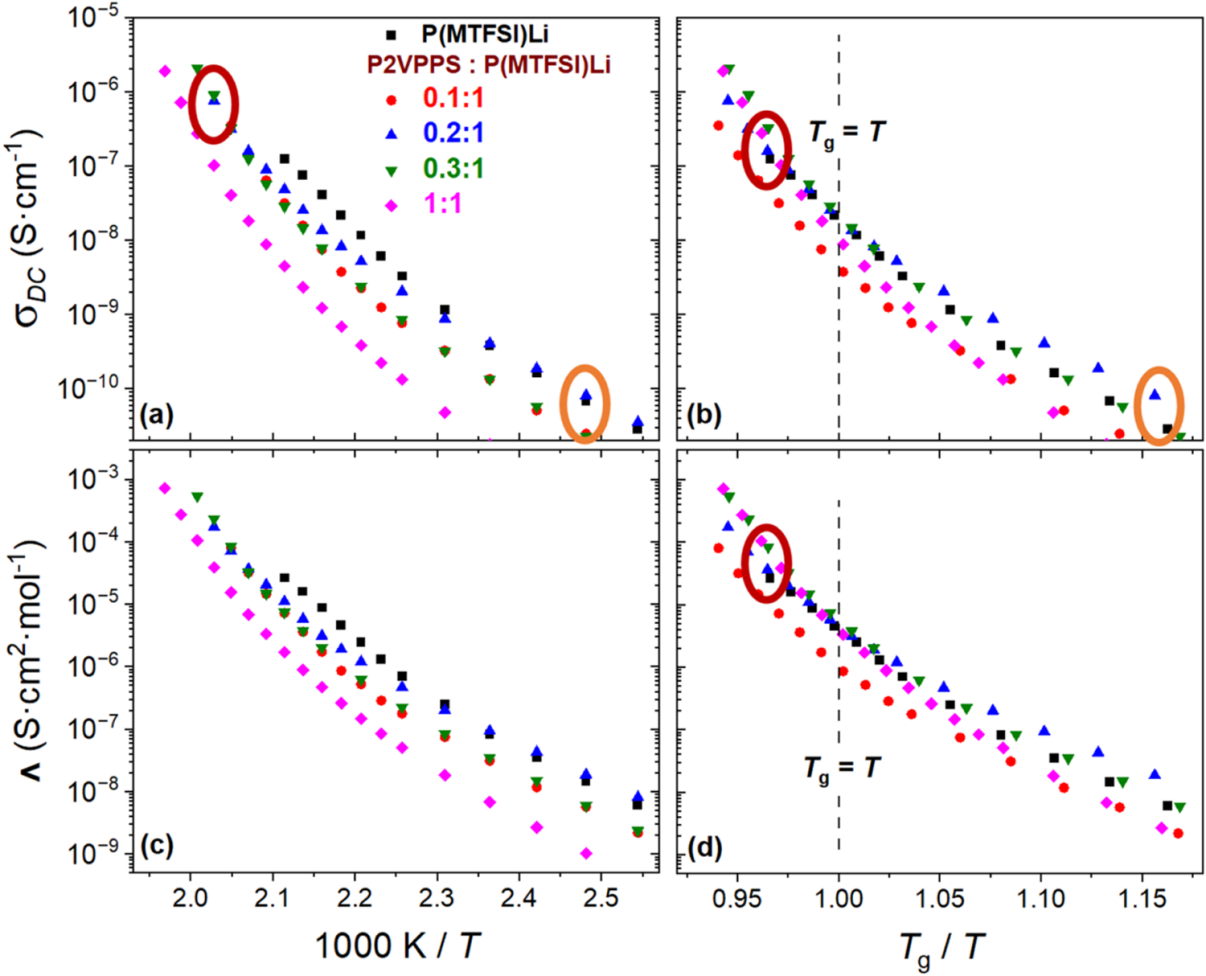
(a) DC conductivity (σ_DC_) as a function of reciprocal temperature and (b) normalized against *T*_g_, corresponding to the molar conductivity (Λ) results in (c) and (d), respectively, for the P2VPPS/P­(MTFSI)Li system. See Figure S10 for the full collection of data. The enhanced conductivities of the 0.2:1 blend in the glassy state (orange ovals) and the 0.3:1 blend in the rubbery state (red ovals) are highlighted.

Further inspection of the conductivity behavior in the glassy state revealed several notable features. First, as the temperature decreases and as *T*_g_/*T* increases, the DC conductivity of the 0.2:1 blend approaches and then surpasses that of neat P­(MTFSI)Li (orange ovals in Figure 5). Specifically at 1000 K /*T* ≈ 2.5 (or *T* = 403 K), the conductivities of the 0.2:1 blend and of neat P­(MTFSI)Li were 8.1 × 10^–11^ and 6.8 × 10^–11^ S·cm^–1^, respectively. As discussed later, we also observe reduced energy barriers for the 0.2:1 blend relative to that of P­(MTFSI)­Li. This suggests that decoupled ion transport occurs in a local environment when the blend is glassy, and the local nanostructure, associated with the interaction between P2VPPS and P­(MTFSI)Li as observed in Figure 3, facilitates the Li^+^ ion hopping between neighboring TFSI^–^ groups.

In contrast, blends in the rubbery state show slightly different behaviors. The highest conductivity among the rubbery blends was observed for blend ratios of 0.3:1 (red ovals in Figure 5), with a ratio of 0.2:1 being the next-highest, although we were not able to measure the conductivity of the neat P­(MTFSI)­Li. Specifically at 1000 K /*T* ≈ 2.0 (or *T* = 493 K), the conductivity of the 0.3:1 blend was 9.0 × 10^–7^ S·cm^–1^. In comparing the DC and molar conductivities against *T*_g_/*T* (≈ 0.96), it is notable that the 0.2:1, 0.3:1, and 1:1 P2VPPS: P­(MTFSI)Li blends all exhibit values above that of neat P­(MTFSI)­Li. Specifically, their corresponding DC conductivities are σ_*DC*_= 1.6 × 10^–7^, 3.2 × 10^–7^, 2.8 × 10^–7^, and 1.2 × 10^–7^ S·cm^–1^ respectively, and their corresponding molar conductivities are Λ = 3.6 × 10^–5^, 8.4 × 10^–5^, 1.0 × 10^–4^, and 2.6 × 10^–5^ S·cm^2^·mol^–1^, respectively. All three of these blends showed evidence of an emergent nanostructure in SANS investigations, as shown in Figure 3. Again, this shows evidence for decoupling of ion transport from the segmental dynamics of the polymer chains.

To further reveal the features of ionic conductivity varying with blend composition, the DC conductivity (σ_DC_) and molar conductivity (Λ) at *T*_g_ and a specific temperature were collected in Figure 6. These data were used to examine the impact of varying molar ratios of P2VPPS to P­(MTFSI)Li on ionic transport in the rubbery state (Figure 6b, *T* = 473 K) and the glassy state (Figure 6c, *T* = 403 K), respectively. It should be noted that even at a high temperature of 473 K, which is slightly above the *T*_g_ (471 K) of the 0.3:1 blend, the 1:1 blend (*T*_g_ = 479 K) remains in the glassy state, resulting in significantly lower conductivity, as shown in Figure 6b. With the addition of P2VPPS, a reduction in the ionic conductivity was initially observed in the 0.1:1 blend, likely due to an increase in the *T*_g_. However, for the 0.2:1 blend, which contains more P2VPPS, the ionic conductivity tended to reach a higher value despite the blend’s higher *T*_g_. This improvement in conductivity may be related to the formation of the nanostructure shown in Figure 3, which facilitates ion transport. For the other component ratios (0.3:1 and 1:1), ionic conductivities remained nearly constant (Figure 6a), and although these values slightly decreased in the glassy state (Figure 6b,c), this reduction can be ascribed to the further decrease in segmental dynamics within the blend. These results suggest that the nonmonotonic changes may be attributed to the combined effects of segmental dynamics and morphology in P2VPPS/P­(MTFSI)Li blends, for which there exists a desirable blend composition that potentially offers improved conductivity. In other words, the competition between segmental dynamics, influenced by the high *T*_g_, and the morphology formed in the P2VPPS/P­(MTFSI)Li blend due to electrostatic interactions could explain the variations observed in Figure 6. Figure S11 illustrates the nonmonotonic changes in conductivity at a fixed distance above or below *T*_g_ (specifically, *T*_g_/*T* = 0.96 and 1.15). The results show that conductivity trends with composition are fairly similar, with conductivity in the blend being suppressed below *T*_g_ (*T*_g_/*T* = 1.15) and enhanced above *T*_g_ (*T*_g_/*T* = 0.96), further emphasizing that ionic conductivity is strongly dependent on *T*_g_. Further discussions are provided below.

**Figure 6:**
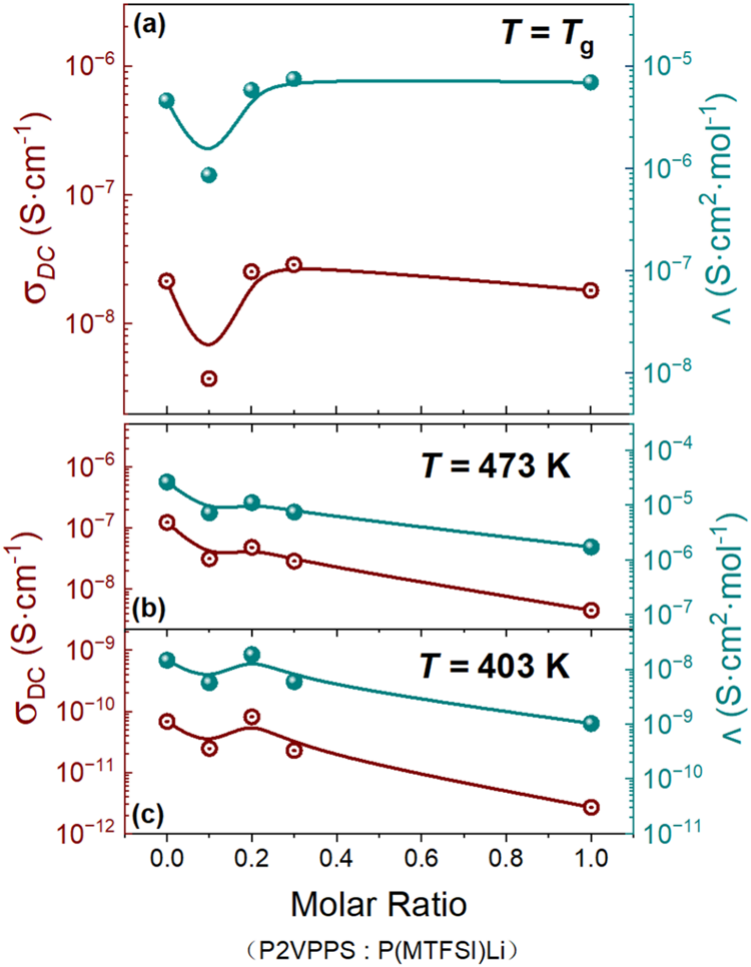
DC conductivity (σ_DC_) and molar conductivity (Λ) at (a) *T* = *T*_g_, (b) *T* = 473 K, and (c) *T* = 403 K as a function of the various mixing molar ratios (P2VPPS: P­(MTFSI)­Li). The σ_DC_ initially decreases due to the increases in the blends’ *T*_g_. The σ_DC_ then increases because of the formation of the local nanostructure. The σ_DC_ decreases once again due to the further increases in the blends’ *T*_g_.

These blends exhibit different ion transport behavior compared to conventional plasticized polymer electrolytes, where it is well documented that ionic conductivity significantly increases as *T*_g_ decreases in plasticized and filler-added polymer electrolytes.^55–57^ These studies particularly highlight the critical role of *T*_g_ in Li^+^ ion mobility within PEG/PEO-based lithium ionic conductors by controlling the segmental motion of polymer chains. Specifically, the decrease in *T*_g_ and the corresponding improvement in conductivity are associated with ion–dipole interactions between lithium ions and plasticizers.^55^ Furthermore, the addition of a plasticizer facilitates segmental chain motion and enhances Li^+^ ion dissociation, leading to a higher level of ion transport. However, in the polyzwitterion/polyanion system, the conductivity behavior becomes more complex and does not simply increase as *T*_g_ decreases. Instead, a nonmonotonic behavior is found. The highest ionic conductivities are observed in the 0.2:1 and 0.3:1 P2VPPS/P­(MTFSI)Li blends, which we hypothesize is attributed to the formation of the local nanostructure. At other compositions, the nanostructure is either not present (or not as prominent) or the *T*_g_ is higher. This can be ascribed to the competition between the slowdown of segmental dynamics (due to the high *T*_g_) and the effect of the local nanostructure formed in the P2VPPS/P­(MTFSI)Li blend. As for the emergent nanostructure, we hypothesize that introduction of the polyzwitterion into the polyanion reduces coordination between the lithium cations and the polyanion’s TFSI group, which competitively interacts with the polyzwitterion’s pyridinium. As a result, the lithium cation experiences easier dissociation from the TFSI^–^ group of P­(MTFSI)­Li, and the ionic conductivity is enhanced. In contrast, the cation–anion interactions between Li^+^ ions and TFSI^–^ groups in neat P­(MTFSI)Li are stronger due to the high charge density of Li^+^, resulting in a higher binding energy. This stronger interaction limits the mobility of the Li^+^ ion, as more energy is required to dissociate ion pairs. Therefore, the morphology generated in P2VPPS/P­(MTFSI)­Li is considered to have a lower energy barrier for lithium cation transport, as each Li^+^ ion has to overcome an attractive cation–dipole interaction potential rather than a cation–anion interaction potential, as in neat P­(MTFSI)­Li.

The nature of these blends implies single-ion conductivity because all anionic groups are covalently tethered to the polymer backbone. Therefore, the majority of mobile charge carriers are assumed to be Li^+^ ions, and a near-unity transference number is expected. Unfortunately, direct measurement of the transference number was not possible because of the low conductivity, high resistance (Figure S12), and the brittle nature of the blends. Transference number measurements are planned for an ongoing and separate study of blends with lower *T*_g_ values and higher conductivities.

In order to gain a deeper understanding of ion transport performance, we analyzed the effective activation energy (E*) at different temperatures for varying P2VPPS/P­(MTFSI)Li blends, as shown in Figure 7. Gainaru et al. reported that the apparent Arrhenius approximation significantly overestimates the energy barrier for ion hopping.^26^ Figure S13 and Table S1 show the results of Arrhenius model fitting of sub-*T*_g_ conductivities yielding temperature-independent activation energy values ranging from 122 to 141 kJ mol^–1^, with the 0.2:1 blend corresponding to the lowest value. The temperature dependence of E* can instead be estimated by an alternative method developed by Gainaru and co-workers:

3 $$E{\;}^{*}=\mathrm{RT}\;\ln\left(\tau /\tau_{0}\right)$$

where E* is the effective activation energy, τ_0_ is a prefactor set to a value of 10^–13^ s, and τ is the temperature-dependent conductivity relaxation time (Figure S14), extracted by using the RBM mentioned above for ionic conductivity data collection. The temperature-dependent energy barriers extracted from the relaxation time match the values obtained from ionic conductivity at a fixed prefactor, providing strong justification for using the Nernst–Einstein formalism to determine energy barriers.^58^ In Figure 7a, E* decreases as the temperature increases for all blends (from about 90–100 to 65 kJ mol^–1^), which corresponds with the enhanced ionic conductivity. Notably, the highest E* is observed in the 1:1 blend, which has the highest *T*_g_ and lowest ionic conductivity (Figure 5) at the same temperature. To better understand the changes in the rubbery and glassy states, *T*_g_-normalized E* is illustrated in Figure 7b. Above *T*_g_ (*T*_g_/*T* < 1), all E* values converge into a similar range, with the lowest value tending to appear in the 0.3:1 blend, indicating that the enhanced ionic conductivity in this region is primarily due to the movement of polymer chains overcoming comparable energy barriers. Below *T*_g_ (*T*_g_/*T* > 1), the lowest E* appears in 0.2:1 blend as marked in orange oval, which is consistent with this blend having the highest ionic conductivity in the glassy state. This further supports the idea that ion transport can be improved even at elevated *T*_g_, in comparison with the neat polyanions.

**Figure 7:**
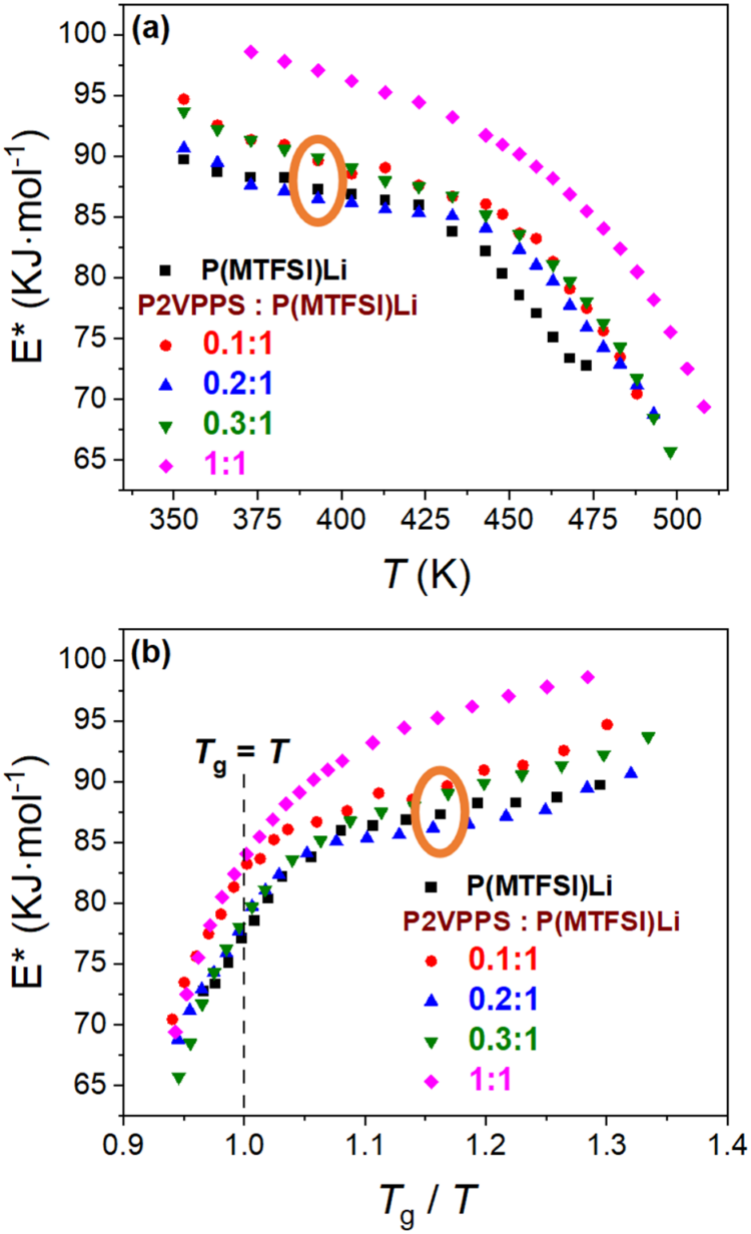
Effective activation energy (E*), as described in eq 3, (a) as a function of temperature and (b) as a function of *T*_g_/*T* for the P2VPPS/P­(MTFSI)Li system, respectively.

## Conclusions

Polyzwitterion and polyanion (P2VPPS/P­(MTFSI)­Li) blends were investigated to understand their thermal behaviors and unique morphologies associated with ionic transport. Neat P2VPPS exhibited a higher glass transition temperature (*T*_g_) of 499 K compared to that of neat P­(MTFSI)Li (457 K). Their blends displayed a single *T*_g_ within the range of the neat polymers, indicating good miscibility and the absence of crystallinity. Furthermore, the *T*_g_ values of blends increased with P2VPPS content, demonstrating restricted P­(MTFSI)Li chain dynamics. Specifically, the *T*_g_ of blends followed the Gordon–Taylor equation, with a positive *k* value (1.83) signifying strong attractive intercomponent interactions. Interestingly, X-ray and neutron scattering revealed a persistent nanostructure (∼24 Å) in the blends across all of the studied temperatures. This ordered local structure was considered to be a key factor in facilitating ionic conductivity. Among the blends, the 0.2:1 composition exhibited the highest DC conductivity (σ_DC_) in the glassy state, whereas the 0.3:1 composition showed the highest σ_DC_ in the rubbery state, both corresponding to the lowest effective activation energy (E*), despite its relatively high *T*_g_. This enhancement was attributed to the facilitation of the Li^+^ ion transport by the nanostructure. Specifically, in the presence of P2VPPS, Li^+^ ions overcame a weaker attractive cation–dipole interaction potential in the P2VPPS/P­(MTFSI)­Li blend rather than the stronger cation–anion interaction potential in neat P­(MTFSI)­Li. However, the conductivities of the 0.3:1 and 1:1 blends were not further enhanced with additional P2VPPS, likely due to the counteracting effect of increased *T*_g_, which slowed segmental dynamics.

These findings provide insights into ionic transport in polyzwitterion/polyelectrolyte blends, highlighting the competing effects of *T*_g_ (limiting segmental mobility) and local nanostructure formation (enhancing ionic conductivity). Although the high *T*_g_ values of the blends led to low room-temperature conductivities, this study points to a promising path forward. That is, blends of polyanion and polyzwitterion with lower *T*_g_ values may be able to preserve the emergent structure while providing increased segmental mobility and therefore higher conductivity. Accordingly, our future research lies in pursuing this direction in achieving an ideal balance of *T*_g_ and nanostructure formation for enhanced conductivity in solid-state electrolytes.

## Supplementary Material



## Data Availability

The data that support the findings of this study are available on Constellation (doi: 10.13139/ORNLNCCS/2529465), a service of the Oak Ridge Leadership Computing Facility at the Oak Ridge National Laboratory, which is supported by the Office of Science of the U.S. Department of Energy under Contract No. DE-AC05–00OR22725.

## Notes

Details of the TGA and synthesis of the deuterated monomer are presented. SAXS/SANS data at different temperatures are presented. Also, BDS data and associated analysis of the data are presented. Furthermore, the coarse-grained model for the molecular dynamics simulations is discussed (PDF)

## Abbreviations

BDS: broadband dielectric spectroscopy
CGMD: coarse-grained molecular dynamics
DC: direct current
DSC: differential scanning calorimetry
FENE: finite extensible nonlinear elastic
LJ: Lennard-Jones
MTFSI-Li: lithium ((3-((2-(methyl-d3)­acryloyl-d2)­oxy)­propyl)­sulfonyl)­((trifluoromethyl)-sulfonyl)­amide
P2VPPS: poly­(1-(3-sulfonatopropyl)-2-vinylpyridinium)
PA: polyanion
PEG: polyethylene glycol
PEO: poly­(ethylene oxide)
P­(MTFSI)­Li: poly­(lithium (trifluoromethane)­sulfonimide methacrylate)
PZ: polyzwitterion
RBM: random barrier model
SANS: small-angle neutron scattering
SNS: spallation neutron source
SPEs: solid polymer electrolytes
SAXS: small-angle X-ray scattering
*T*_d_: decomposition temperature
*T*_g_: glass transition temperature
TGA: thermogravimetric analysis
E*: effective activation energy
*k*: a constant in Gordon–Taylor equation
*q*: magnitude of scattering vector
*U*_LJ_: LJ potential
*U*_elec_: coulomb potential
*w*_1_ and *w*_2_: weight fractions of each component in blend
ϵ_0_: permittivity of vacuum
σ_DC_: DC conductivity
Λ: molar conductivity
τ_LJ_: LJ time
τ_0_: a prefactor set to a value of 10^–13^ s
τ: relaxation time
