# Supplementary material for: Emergent Nanostructure and Ion Transport in Polyzwitterion/Polyanion Blends
Source: Macromolecules. 2025 Aug 13;58(16):8658–69. doi: 10.1021/acs.macromol.5c00806 (PMC12392716; doi:10.1021/acs.macromol.5c00806)
Supplement: Supplementary file 1 [file ma5c00806_si_001.pdf]

Supporting Information

Emergent Nanostructure and Ion Transport in Polyzwitterion/Polyanion Blends

Hongwei Li,[1] Qinyu Zhu,[2] Yuya Shinohara,[3] Yangyang Wang,[2] Panagiotis Christakopoulos,[2] Autumn F. Kudlack,[1] Zitan Huang,[4] Peter V. Bonnesen,[2] Changwoo Do,[5] Md Anisur Rahman,[6] Michelle L. Lehmann,[6] Tomonori Saito,[6] Ralph H. Colby,[4] Rajeev Kumar,[2] Jodie L. Lutkenhaus[1,7]**

1. Artie McFerrin Department of Chemical Engineering, Texas A&M University, College Station, Texas 77843, United States
2. Center for Nanophase Materials Sciences, Oak Ridge National Laboratory, Oak Ridge, Tennessee 37831, United States
3. Materials Science and Technology Division, Oak Ridge National Laboratory, Oak Ridge, Tennessee 37831, United States
4. Department of Materials Science and Engineering, The Pennsylvania State University, University Park, Pennsylvania 16802, United States
5. Neutron Scattering Division, Oak Ridge National Laboratory, Oak Ridge, Tennessee 37831, United States
6. Chemical Sciences Division, Oak Ridge National Laboratory, Oak Ridge, Tennessee 37830, United States
7. Department of Materials Science and Engineering, Texas A&M University, College Station, Texas 77840, United States

Corresponding email: kumarr@ornl.gov and jodie.lutkenhaus@tamu.edu

Additional Materials & Methods

S1. Nuclear Magnetic Resonance (NMR). NMR spectra were obtained at the Center for Nanophase Materials Sciences on a Bruker Avance NEO NMR console coupled to a 11.74 T actively shielded magnet (Magnex Scientific/Varian) operating at 499.717 MHz for proton. All spectra were acquired at 298 K in either D_2O (4.80 ppm ^1H reference), $CDCl_3$ (7.27 ppm ^1H reference and 77.23 ppm ^{13}C reference) or DMSO-d_6 (2.50 ppm ^1H reference and 39.51 ppm ^{13}C reference). Assignments were confirmed using a combination of proton, COSY, carbon, carbon APT and HSQC experiments.

S2. Synthesis of Deuterated Monomer

Reaction scheme used for synthesis of the deuterated monomer is shown in Scheme S1. Details of synthesis and chemical characterization of various reagents involved in this synthesis scheme are presented below.

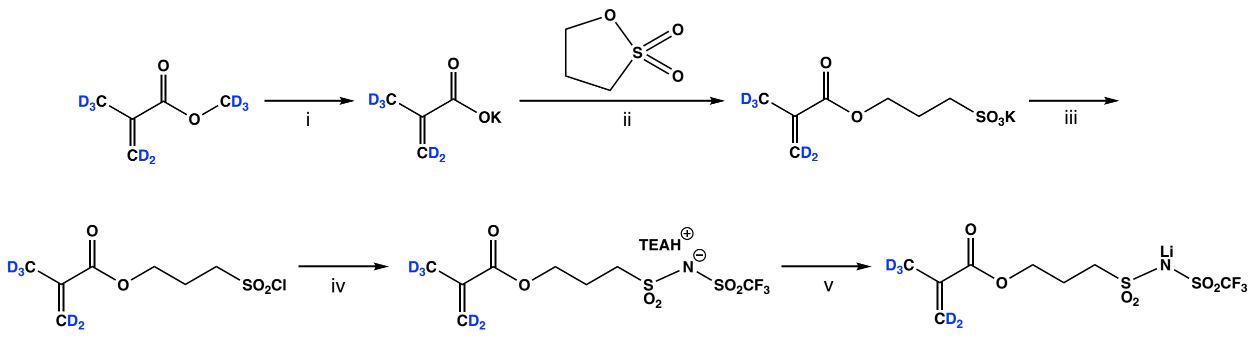

Scheme S1. Reagents and conditions: (i) KOH, D_2O, 273 K to room temperature (RT), 21 h, quant.; (ii) CH_3CN, MEHQ, 353 K, 8 d, quant.; (iii) thionyl chloride, 16:1 v/v THF:DMF, 273 K

for 1.3 h, RT for 19.5 h, near quant.; (iv) $CF_3SO_2NH_2$, TEA, THF, 273 K for 1h, RT for 21 h, 78%; (v) LiH, THF, 273 K to RT, 3 h, 89%.

Materials. Methyl-d_3 methacrylate-d_5 (≥99 atom%D, ≥99% CP) was obtained from Millipore Sigma.

Potassium 2-(methyl-d_3)acrylate-d_2

In a 250-mL round bottom flask, 5.19 g of KOH (99.99% trace metal purity, nominally 85% chemical purity) was dissolved in 35 mL of D_2O, 99.8 atom% D) with stirring in an ice water bath. About 2 mg of hydroquinone was added, followed by the dropwise addition of methyl-d_3 methacrylate-d_5 (10.0 g, 92.4 mmol). The flask was stoppered and stirring continued for 5 min, whereupon the ice-bath was removed and stirred solution allowed to warm to ambient temperature. The solution was visually inspected to determine whether all the of methyl-d_3 methacrylate-d_5 had reacted, and additional KOH (total 0.949 g) was added in five small portions over the course of 3 hours, at which point the solution was clear with no signs of suspended droplets of methyl-d_3 methacrylate-d_5, indicating a KOH chemical purity of 84.5%. The solution was allowed to stir for an additional 17 h, after which the D_2O was removed and recovered by rotary evaporation at 323 K. The off-white solid was dried overnight under vacuum to afford the product in quantitative yield. $^{13}C\{^1H\}$ NMR (D_2O): δ 177.5 (s, *C*=O), 142.1 (s, *C*=CD_2), 119.9 (pent, J_{CD} = 23.8 Hz), C=*C*D_2), 18.1 (sept, J_{CD} = 19.7 Hz), –*C*D_3).

Potassium 3-((2-(methyl-d_3)acryloyl-d_2)oxy)propane-1-sulfonate

In a 500-mL round bottom flask potassium 2-(methyl-d_3)acrylate-d_2 (10.98 g, 85.0 mmol) was suspended in 200 mL dry CH_3CN with a stirbar and MEHQ (210 mg, 1.69 mmol, 2% on monomer). A solution of propane sultone (11.94 g, 97.8 mmol) dissolved in 50 mL dry CH_3CN

was added, and the solution heated to gentle reflux with rapid stirring. The reaction continued in this manner for 8 days, during which the consistency became very thick and creamy. After cooling, the CH_3CN was removed by rotary evaporation at 313 K to afford a moist off-white solid, which was dried overnight under high vacuum to a white powder. NMR analysis in D_2O shows the product plus some excess unreacted propane sultone. The product was washed with diethyl ether (4 x 150 mL) to remove the propane sultone, and filtered with suction, followed by drying overnight under high vacuum. The final yield is 21.4 g (quantitative). ^{1}H NMR (D_2O): δ 4.34 (br t, J = 6.3 Hz, 2H, –OCH_2CH$_2$–), 3.096/3.084 (br overlapping d, J_{Ha} = 9.7 Hz, J_{Hb} = 9.5 Hz, 2H, –CH_aH_bSO$_3$K), 2.21 (m, 2H, –OCH$_2$CH_2–). ^{13}C{^{1}H} NMR (D_2O): δ 169.8 (s, *C*=O), 135.6 (s, *C*=CD$_2$), 126.3 (pent, J_{CD} = 24.2 Hz, C=*C*D$_2$), 63.7 (s, –O*C*H$_2$CH$_2$–), 47.8 (s, –*C*H$_2$SO$_3$K), 23.7 (s, –OCH$_2$*C*H$_2$–), 16.5 (sept, J_{CD} = 19.7 Hz), –*C*D$_3$).

3-(chlorosulfonyl)propyl 2-(methyl-*d*$_3$)acrylate-*d*$_2$

In a 100-mL round bottom flask potassium 3-((2-(methyl-*d*$_3$)acryloyl-*d*$_2$)oxy)propane-1-sulfonate (12.57 g, 50.0 mmol) was suspended in 25 mL dry THF with a stir bar, along with 1.5 mL dry DMF. A pressure-equalizing dropping funnel was attached, and the slurry was cooled in an ice-water bath under nitrogen flow. The funnel was charged with thionyl chloride (18.2 mL, 250 mmol), which was added dropwise under nitrogen to the stirred slurry at 273 K over the course of 20 min. Stirring was continued for 1 h at 273 K, whereupon the cold bath was removed and the now turbid yellow solution allowed to warm to ambient temperature. Stirring was continued for an additional 19.5 h, after which the reaction mixture was poured into a beaker containing 150 mL of ice-water. The entire mixture was transferred to a separatory funnel and extracted with dichloromethane (2 x 80 mL). About 2 mg MEHQ was added to the combined dichloromethane extracts, which were washed once with DI water

(50 mL). After drying through a column of granular anhydrous sodium sulfate, the solvent was removed by rotary evaporation at 295 K. The obtained yellow oil was dried briefly under high vacuum to afford 11.82 g of a light amber oil. Proton NMR analysis revealed the presence of residual DMF at 4 moles%, indicating a near quantitative yield of material. The oil was stored at 262 K until ready to use in the subsequent step. ^{1}H NMR ($CDCl_3$): δ 4.32 (br t, J = 6.1 Hz, 2H, –O$CH_2$$CH_2$–), 3.792/3.780 (br overlapping d, J_{Ha} = 9.6 Hz, J_{Hb} = 9.7 Hz, 2H, –C$H_aH_b$$SO_2Cl$), 2.43 (m, 2H, –O$CH_2$$CH_2$–). ^{13}C{^{1}H} NMR ($CDCl_3$): δ 167.1 (s, *C*=O), 135.6 (s, *C*=CD_2), 126.0 (pent, J_{CD} = 24.5 Hz, C=CD_2), 62.4 (s, –O$CH_2$$CH_2$–), 61.4 (s, –$CH_2SO_2Cl$), 24.4 (s, –O$CH_2$$CH_2$–), 17.5 (sept, J_{CD} = 19.3 Hz), –CD_3).

Triethylammonium ((3-((2-(methyl-d_3)acryloyl-d_2)oxy)propyl)sulfonyl)((trifluoromethyl)sulfonyl) amide

Trifluoromethanesulfonamide (7.36 g, 49.4 mmol) and triethylamine (15.25 mL, 110 mmol) were dissolved in 35 mL dry THF in a 100-mL round bottom flask with stir bar. A pressure-equalizing dropping funnel was attached, and the stirred solution cooled in an ice-water bath under nitrogen. The funnel was charged with the 3-(chlorosulfonyl)propyl 2-(methyl-d_3)acrylate-d_2 from the previous reaction diluted with 16.5 mL dry THF, which was added dropwise under nitrogen to the stirred reaction mixture at 273 K over the course of 25 min. Stirring was continued for 1 h at 273 K, whereupon the cold bath was removed and the turbid light brown suspension allowed to warm to ambient temperature. Stirring was continued for an additional 21 h, after which the suspension was filtered through a sintered glass frit into a receiver flask containing 1 mg of BHT. The volatiles were removed from the filtrate by rotary evaporation at 292 K to afford an amber oil, which was diluted with dichloromethane (100 mL). The dichloromethane solution was washed with DI water (4 x 35 mL) and dried through

a column of granular anhydrous sodium sulfate. Additional BHT (1 mg) was added, and the solvent was removed by rotary evaporation at 289 K - 291 K. THF (5 mL) was added, and the volatiles removed again via rotary evaporation at 291 K. This monomer tends to auto-polymerize if dried down too hard, so after briefly drying under high vacuum, and removing a sample for NMR analysis, the amber oil (ca 25 g) contained in a 250-mL round bottom flask was further diluted with THF (10 mL) and the solution stored at 262 K until the next step. Proton NMR analysis, which shows THF and dichloromethane residues, indicates the product yield at ca 17.2 g (78%), with fluorine NMR indicating a purity of 95.5%. ^{1}H NMR ($CDCl_3$): δ 8.09 (v. br s, 1H, TEA*H*$^+$), 4.24 (br t, J = 6.2 Hz, 2H, –OC*H*$_2$CH$_2$–), 3.252/3.240 (br overlapping d, J_{Ha} = 9.4 Hz, J_{Hb} = 9.4 Hz, 2H, –C*H*$_a$*H*$_b$SO$_2$N), 3.18 (q, J = 7.3 Hz, 6H, –C*H*$_2$– of TEAH$^+$), 2.21 (m, 2H, –OCH$_2$C*H*$_2$–), 1.33 (t, J = 7.3 Hz, 9H, –C*H*$_3$ of TEAH$^+$). ^{13}C{^{1}H} NMR ($CDCl_3$): δ 167.4 (s, *C*=O), 135.9 (s, *C*=CD$_2$), 125.4 (pent, J_{CD} = 24.9 Hz, C=*C*D$_2$), 120.3 (q, J_{CF} = 323 Hz, –*C*F$_3$), 62.8 (s, –O*C*H$_2$CH$_2$–), 52.0 (s, –*C*H$_2$SO$_2$N), 46.8 (s, –*C*H$_2$– of TEAH$^+$), 23.8 (s, –OCH$_2$*C*H$_2$–), 17.5 (sept, J_{CD} = 19.8 Hz), –*C*D$_3$), 8.7 (s, –*C*H$_3$ of TEAH$^+$). ^{19}F NMR ($CDCl_3$): δ -78.0 (s, –C*F*$_3$, 95.5%), with an impurity peak at -79.4 (4.5%).

Lithium ((3-((2-(methyl-*d*$_3$)acryloyl-*d*$_2$)oxy)propyl)sulfonyl)((trifluoromethyl)sulfonyl)amide

The 250-mL round bottom flask containing the product above in ca 10 mL THF was removed from cold storage, a stir bar added, and the solution further diluted with dry THF (40 mL). The stirred solution was cooled to 273 K in an ice water bath under nitrogen flow. Lithium hydride powder (0.91 g, 114 mmol, 95% purity) was weighed out in a vial inside an Argon dry box and diluted with dry THF (30 mL), forming a gray suspension. The capped vial was brought out of the dry box, and the LiH-THF suspension added dropwise to the stirred solution at 273 K under nitrogen over the course of 3 min. Hydrogen evolution was observed. The reaction mixture

was stirred at 273 K for an additional 10 min, whereupon then cold bath was removed, and the reaction allowed to warm to ambient temperature. After 3 h, the tan suspension was filtered through a pad of Celite 545 into a receiver flask containing 1 mg BHT. The Celite pad was washed with dry THF (2 x 10 mL), and the filtrate concentrated by rotary evaporation at 291 K to afford a yellow oil. The oil was washed with dry *n*-hexane (3 x 30 mL), decanting off the hexane after each wash. The yellow oil was then diluted with dichloromethane (20 mL), and this suspension washed with additional dry *n*-hexane (2 x 50 mL), decanting the supernatant solvent away from the oil. The yellow oil was dried at ambient temperature first by rotary evaporation then briefly by high vacuum. The mass of the congealed oil at this stage was 17.5 g. A sample was retrieved for NMR analysis, and then the rest stored at 262 K. Prior to polymerization, the semi-solid oil was further dried under vacuum to afford 13 g of a tan foamy solid. Proton NMR analysis, which shows THF, dichloromethane and hexane residues, indicates the product yield at ca 12.0 g (89% from above), with fluorine NMR indicating a purity of 93.7%. ^{1}H NMR (DMSO-d_6): δ 4.17 (br t, J = 6.4 Hz, 2H, –OCH_2CH$_2$–), 3.065/3.053 (br overlapping d, J_{Ha} = 9.2 Hz, J_{Hb} = 9.1 Hz, 2H, –CH_aH_bSO$_2$N), 2.01 (m, 2H, –OCH$_2$CH_2–). ^{13}C{^{1}H} NMR (DMSO-d_6): δ 166.5 (s, *C*=O), 135.6 (s, *C*=CD$_2$), 125.3 (pent, J_{CD} = 24.7 Hz, C=*C*D$_2$), 120.2 (q, J_{CF} = 325 Hz, –*C*F$_3$), 62.7 (s, –O*C*H$_2$CH$_2$–), 51.3 (s, –*C*H$_2$SO$_2$N), 23.6 (s, –OCH$_2$*C*H$_2$–), 17.1 (sept, J_{CD} = 19.5 Hz), –*C*D$_3$). ^{19}F NMR (DMSO-d_6): δ -77.6 (s, –C*F*$_3$, 93.7%), with a impurity peaks at -77.0 (1.3%) and -78.6 (5.0%).

S3. Decomposition temperature (T_d). Thermogravimetric analysis (TGA) was performed on a TA Instruments TGA 5500 using a heating rate of 10 K/min from 295 K to 873 K under a flowing nitrogen atmosphere. Samples with a typical mass of 2.0–5.0 mg were added on platinum HT pan. The decomposition temperature (T_d) was determined from the point on the curve where 95 wt% of mass loss had occurred after solvent (water) evaporation.

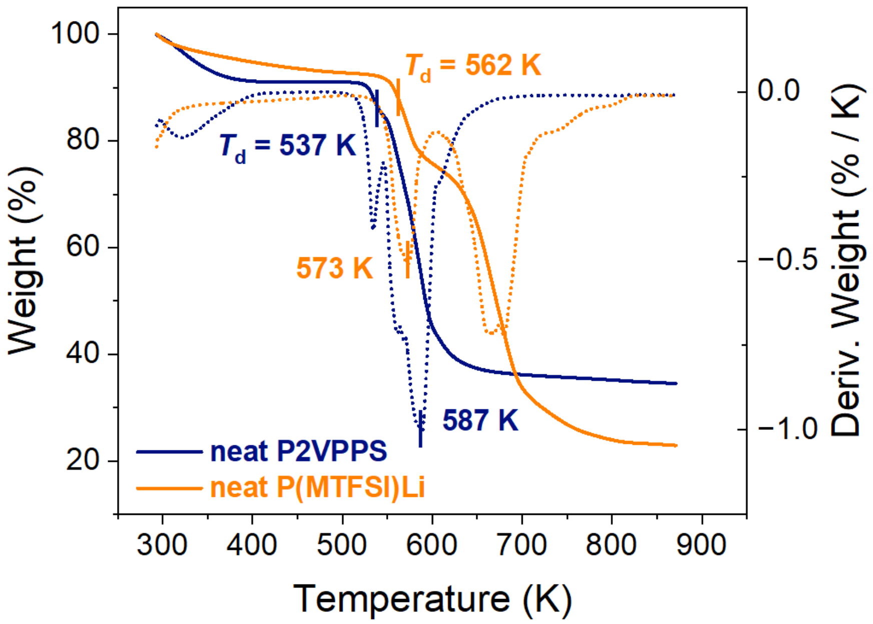

Figure S1. TGA curves of weight percent (solid line) and the corresponding derivative curves (short dot line) for neat polyzwitterion (P2VPPS) and neat polyanion (P(MTFSI)Li).

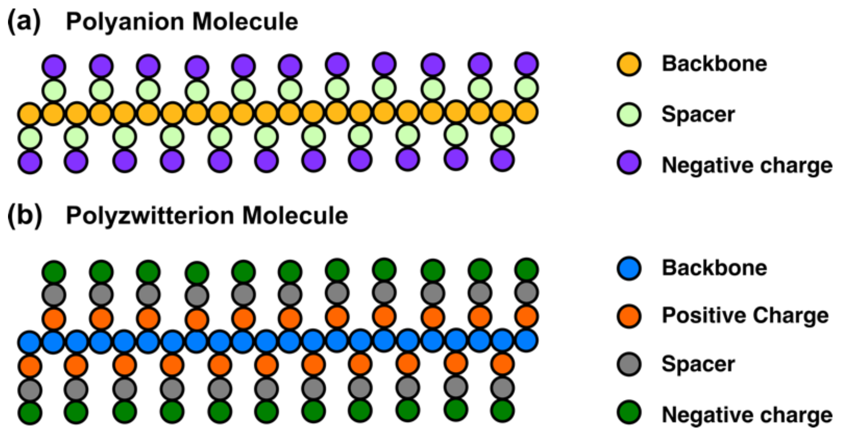

Figure S2. Coarse-grained representation of (a) polyanion and (b) polyzwitterion chains used for molecular dynamics simulations. Positively charged cations, which are the counterions of the polyanion chain, are not shown here

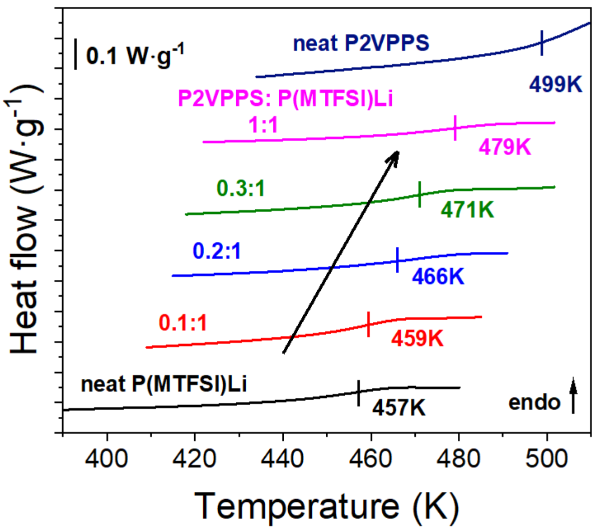

Figure S3. DSC thermograms of neat P2VPPS, neat P(MTFSI)Li, and their blends. The glass transition temperature (T_g) values are marked by short vertical lines. The ratio represented as "a:b" indicates "a" molar repeat units of P2VPSS and "b" molar repeat units for P(MTFSI)Li.

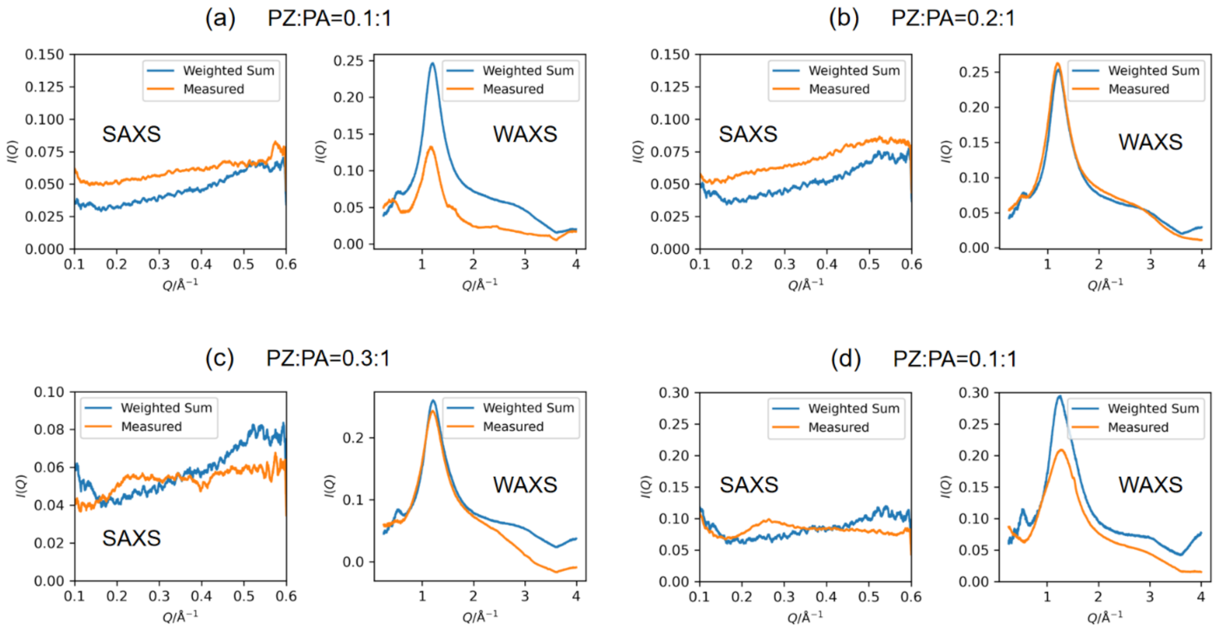

Figure S4. The weighted sum of SAXS/WAXS intensity profiles for (a) PZ:PA=0.1:1, (b) PZ:PA=0.2:1, (c) PZ:PA=0.3:1 and (d) PZ:PA=1:1.

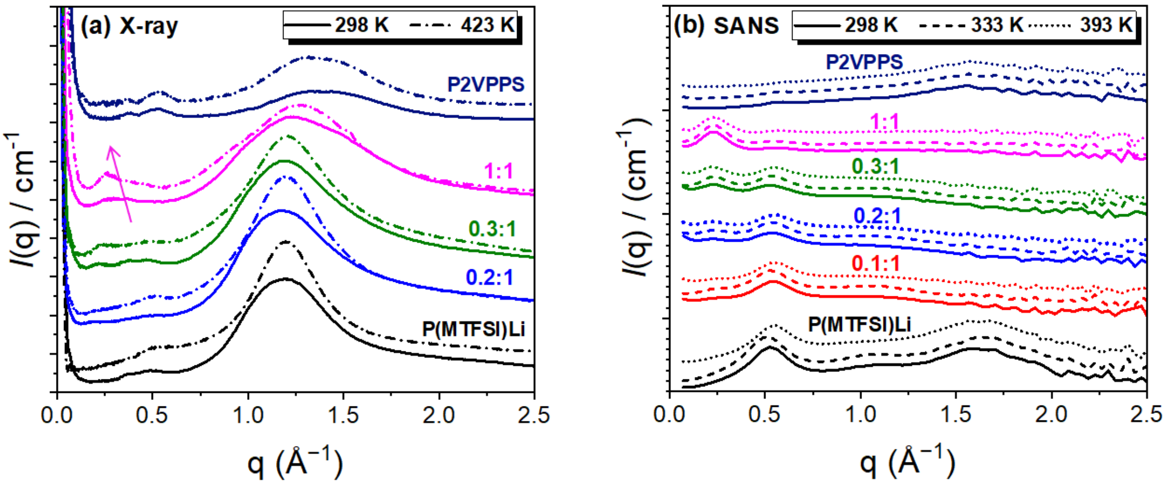

Figure S5. Effects of mixing and temperature (a) 298 K (solid line) and 423 K (shot dash dot) on X-ray and (b) 298 K (solid line), 333 K (shot dash) and 393 K (shot dot) on SANS from P2VPPS/P(MTFSI)Li blends. The intensity profiles were offset vertically by 0.1 cm^{-1} (SANS) and 0.05 cm^{-1} (X-ray), respectively, for clarity and the blend ratio is provided in the figures.

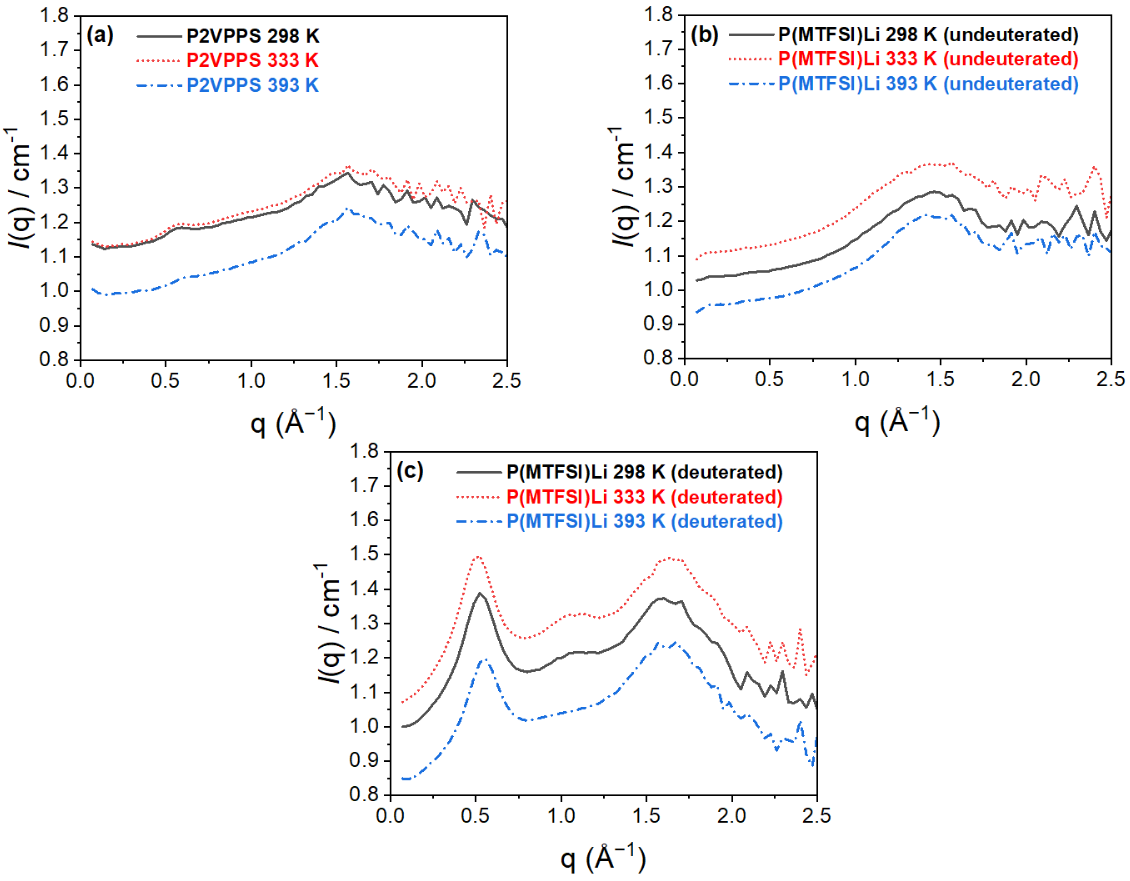

Figure S6. Effects of temperature on SANS from homopolymers (a) neat P2VPPS, (b) neat undeuterated P(MTFSI)Li and (c) neat deuterated P(MTFSI)Li, respectively.

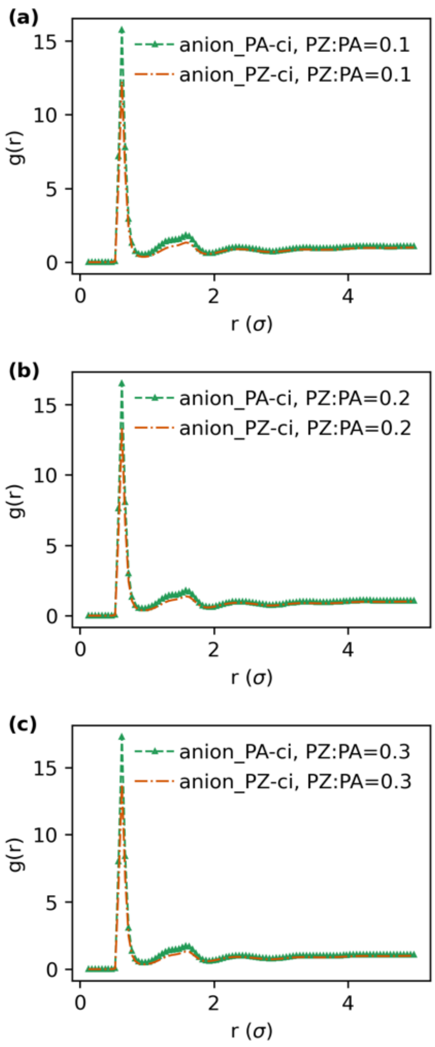

Figure S7. Radial distribution functions of the lithium cation/counterion of the PA anion and PA anion along with the radial distribution functions of the same counterion and anions of the PZs are shown in the blends with mixing ratios of (a) PZ:PA=0.1, (b) PZ:PA=0.2, and (c) PZ:PA=0.3. Strength of the first peak in these radial distribution functions is smaller for the counterion-PZ anion than the counterion-PA anion, which highlights weaker attractive interactions for the counterion-PZ anion than the counterion-PA anion.

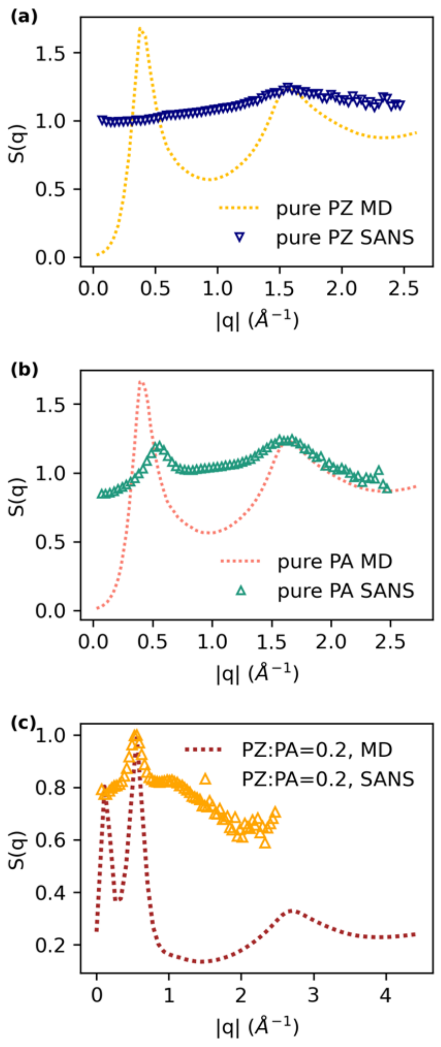

Figure S8. Comparison of structures from the SANS measurements and the CGMD simulations for (a) the pure PZ melt, (b) the pure PA melt, and (c) the blend at PZ:PA=0.2. We rescaled peak 1 in Figure 4a to match the location and intensity of the SANS peak at |q|~1.5 Å^{-1}.for the pure components in (a) and (b), and rescaled peak 2 in Figure 4a to match the SANS peak at |q|~0.5 Å^{-1} for the blend system in (c).

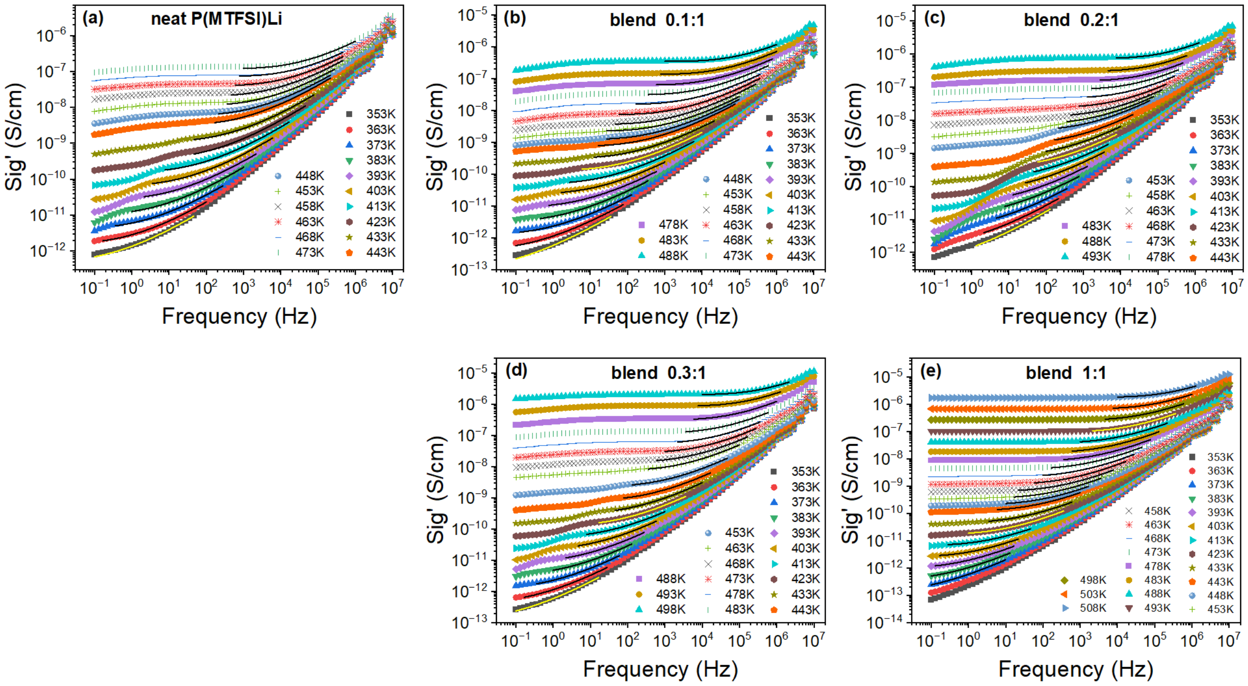

Figure S9. The frequency dependence of the in-phase part of the conductivity (Sig′) responses for (a) neat P(MTFSI)Li and P2VPPS/P(MTFSI)Li blend at (b) 0.1:1, (c) 0.2:1, (d) 0.3:1 and (e) 1:1, respectively. The solid lines are the RBM fittings to collect DC conductivity data.

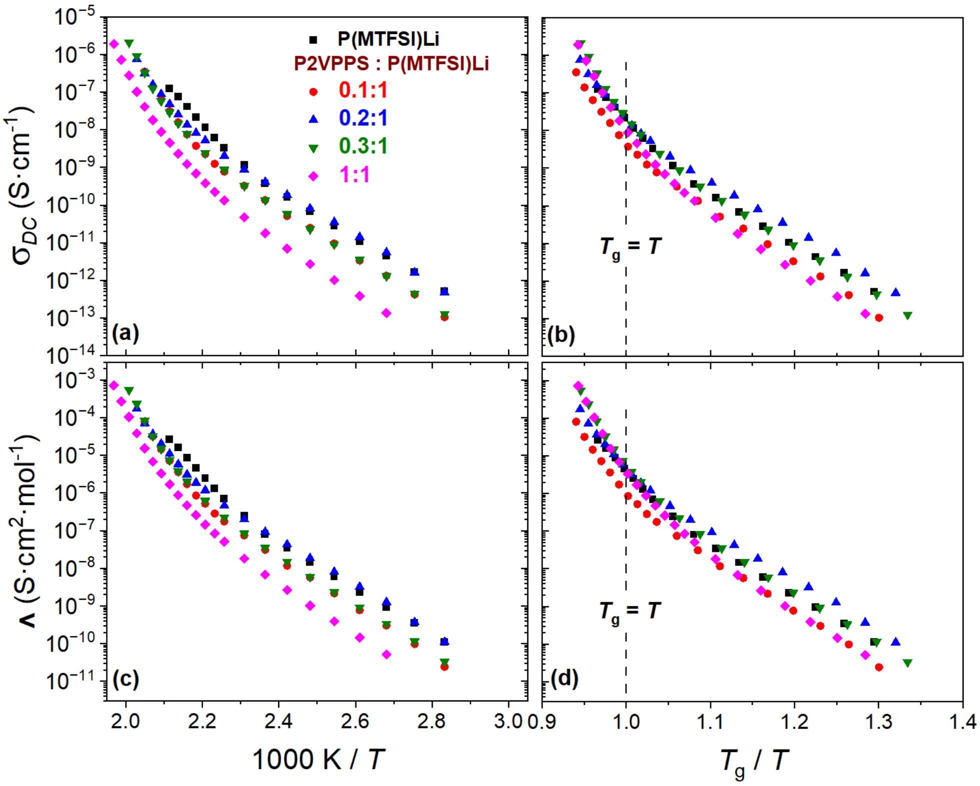

Figure S10. Full data of (a) The DC conductivity (σ_{DC}) as a function of reciprocal temperature and (b) normalized against T_g, corresponding to the molar conductivity (Λ) results in (c) and (d), respectively, for the P2VPPS/P(MTFSI)Li system. See **Figure 5** in the main text for a zoom-in of the data near the T_g.

Molar conductivity (ʌ) calculation based on Li^+ concentration:

We assume that (1) polyzwitterion (P2VPPS) and polyanion [P(MTFSI)Li] are mixed well with different molar ratios of repeat unit; (2) the molar conductivity (ʌ) is calculated according to the lithium ion (Li^+) concentration. The calculation example is as follows: P2VPPS: 227.3 g/mol and P(MTFSI)Li= 333.2 g/mol

For neat P(MTFSI)Li film:

Thickness: 0.0381 cm   Diameter= 0.63 cm

So, the volume of neat P(MTFSI)Li film = $\pi*(0.63/2)^2$ *0.0381 =0.01188 cm^3

The measured mass of P(MTFSI)Li film is 18.5 mg

So, the mole of Li^+ = 18.5*0.001/333.2=0.00005552 mol

Then, molar conductivity (ʌ) is:

$ʌ= \boldsymbol{\sigma_{DC}}$ / (0.00005552/0.01188) $S·cm^2·mol^{-1}$

For blend film of P2VPPS: P(MTFSI)Li = 0.3:1:

Thickness: 0.03429 cm   Diameter= 0.63 cm

So, the volume of blend film = $\pi*(0.63/2)^2$ * 0.03429 = 0.01069 cm^3

The measured mass of P2VPPS/P(MTFSI)Li blend film is 16.5 mg.

So, the mole of Li^+ = 16.5*0.001/(0.3*227.3+333.2)=0.00003514 mol

Then, molar conductivity (ʌ) is:

$ʌ= \boldsymbol{\sigma_{DC}}$ / (0.00003514/0.01069) $S·cm^2·mol^{-1}$

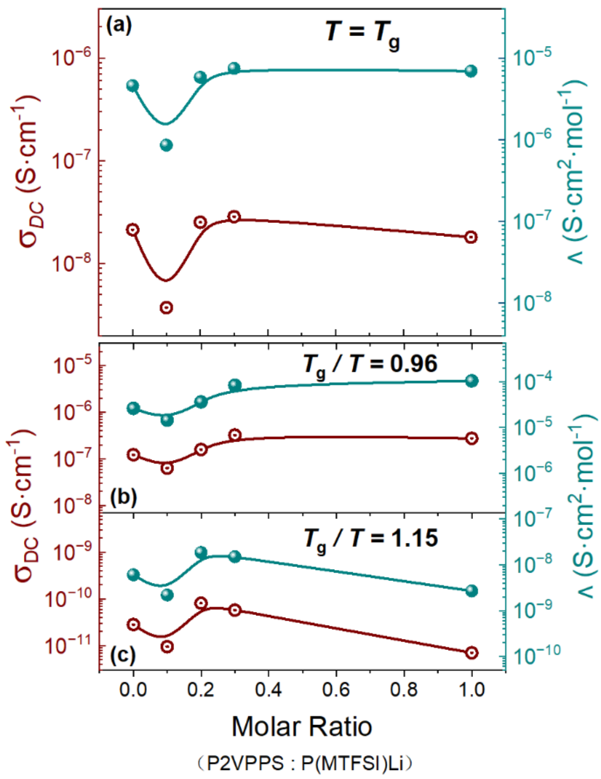

Figure S11. The DC conductivity (σ_{DC}) and molar conductivity (ʌ) at (a) T=T_g, (b) T_g / T = 0.96 and (c) T_g / T = 1.15 as a function of the various mixing molar ratios (P2VPPS : P(MTFSI)Li). The σ_{DC} initially decreases due to the blends' increased T_g. The σ_{DC} then increases because of the formation of the local nanostructure. When below T_g (T_g / T = 1.15), the σ_{DC} decreases once again suggesting that σ_{DC} is suppressed in glassy state, due to the further increases in the blends' T_g.

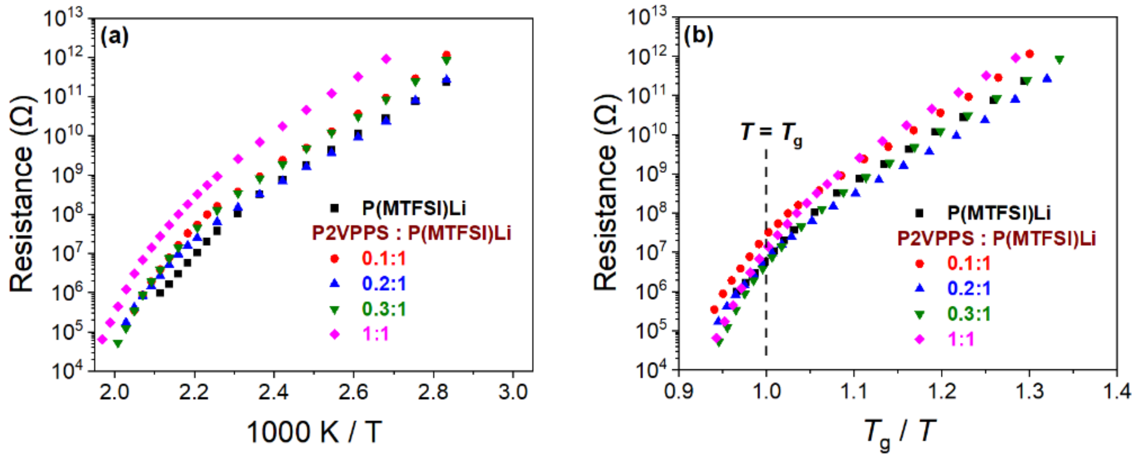

Figure S12. (a) Resistance (**Ω**) (a) as a function of temperature and (b) as a function of Tg/T for the P2VPPS/P(MTFSI)Li system, respectively.

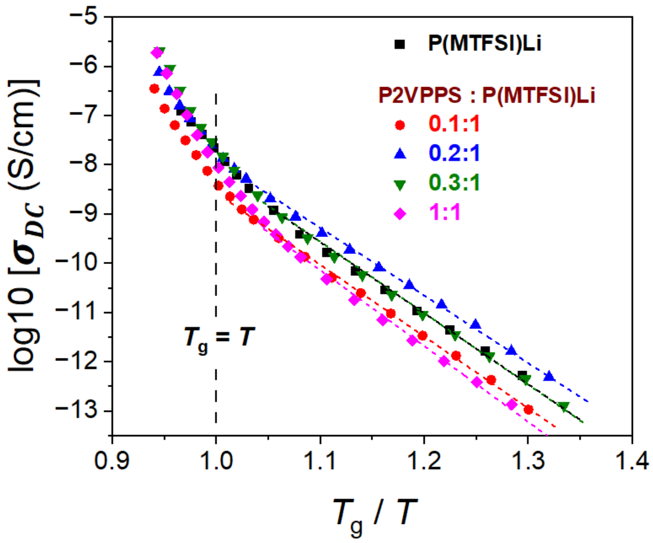

Figure S13. Arrhenius fitting (short dash lines) of T_g normalized conductivity for different compositions of P2VPPS/P(MTFSI)Li. VFT fitting was attempted for data above the T_g, but the fits were unphysical, given the limited range of data due to proximity to the polymer blends' degradation temperature.

Table S1. Fitting parameters for Eq. S1 (Arrhenius) of σ_{DC} data in **Figure S13**.

Molar ratios	σ_{DC} (T_g) [S/cm]	Arrhenius	
		$\log 10\,(\sigma_0)$ [S/cm]	E_a [kJ/mol]
0	2.14×10^{-8}	6.3	126
0.1:1	3.73×10^{-9}	6.0	128
0.2:1	2.53×10^{-8}	5.8	122
0.3:1	2.86×10^{-8}	6.4	130
1:1	1.81×10^{-8}	6.8	141

To further analyze the blends behaviors for ionic transport, we employed an Arrhenius model, which is commonly used to model the temperature dependence of conductivity in materials, suggesting a simple decoupled hopping mechanism independent of the polymer chain motions when a linear Arrhenius variation exists (below T_g).[1-3] The equation is expressed as:

$\sigma_{DC} = \sigma_0 \exp\left(\frac{-E_a}{RT}\right)$ (Eq. S1)

where σ_0 is a pre-exponential factor and E_a represents the activation energy for conductivity corresponding to the slope of logσ_{DC} versus 1/T plots. For the 0.2:1 blend, Arrhenius behavior is observed, corresponding to an activation energy (E_a) of approximately 122 kJ mol^{-1}, which is higher than that of neat P(MTFSI)Li ($E_a \approx 126$ kJ mol^{-1}) in the glassy state (T_g/T >1). This suggests that decoupled ion transport occurs in a local environment when the blend is glassy, and the local nanostructure, associated with the interaction between P2VPPS and P(MTFSI)Li as observed in **Figure 3**, facilitates the Li^+ ion hopping between neighboring $TFSI^-$ groups.

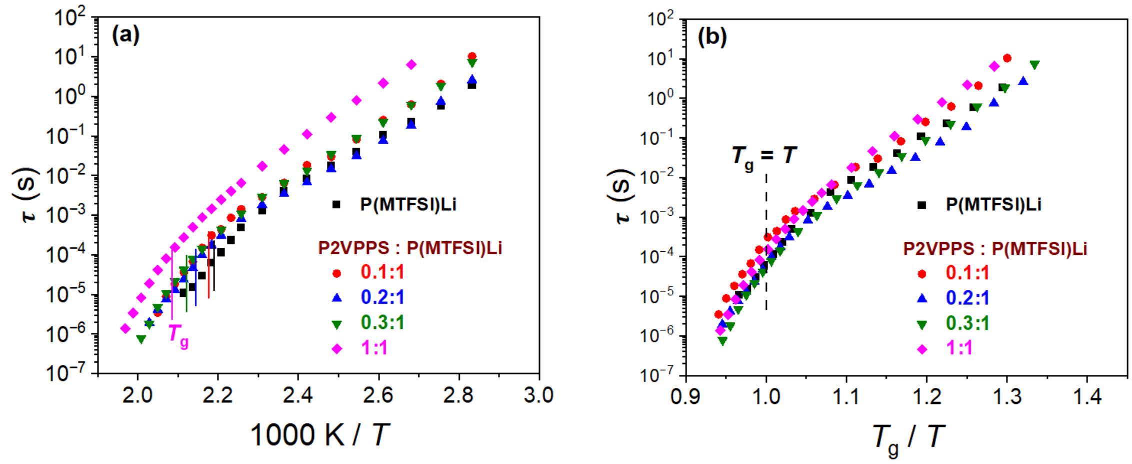

Figure S14. (a) Relaxation time of neat P(MTFSI)Li and blend with P2VPPS at different molar ratios as a function of temperature. (b) T_g-normalized relaxation time of neat P(MTFSI)Li and P2VPPS/P(MTFSI)Li blends

References:

1. Choi, U. H.; Price Jr, T. L.; Schoonover, D. V.; Xie, R.; Gibson, H. W.; Colby, R. H., Role of Chain Polarity on Ion and Polymer Dynamics: Molecular Volume-Based Analysis of the Dielectric Constant for Polymerized Norbornene-Based Ionic Liquids. *Macromolecules* **2020**, *53* (23), 10561-10573.
2. Paren, B. A.; Nguyen, N.; Ballance, V.; Hallinan, D. T.; Kennemur, J. G.; Winey, K. I., Superionic Li-Ion Transport in a Single-Ion Conducting Polymer Blend Electrolyte. *Macromolecules* **2022**, *55* (11), 4692-4702.
3. Gainaru, C.; Kumar, R.; Popov, I.; Rahman, M. A.; Lehmann, M.; Stacy, E.; Bocharova, V.; Sumpter, B. G.; Saito, T.; Schweizer, K. S.; Sokolov, A. P., Mechanisms Controlling the Energy Barrier for Ion Hopping in Polymer Electrolytes. *Macromolecules* **2023**, *56* (15), 6051-6059.
